# Subinhibitory concentrations of glabridin from *Glycyrrhiza glabra L*. reduce *Listeria monocytogenes* motility and hemolytic activity but do not exhibit antimicrobial activity

**DOI:** 10.3389/fmicb.2024.1388388

**Published:** 2024-07-17

**Authors:** Chengshui Liao, Chuan Yu, Jinxiang Guo, Mengxiang Guan

**Affiliations:** ^1^College of Animal Science and Technology/Laboratory of Functional Microbiology and Animal Health, Henan University of Science and Technology, Luoyang, China; ^2^Luoyang Key Laboratory of Live Carrier Biomaterial and Animal Disease Prevention and Control, Luoyang, China; ^3^The Key Lab of Animal Disease and Public Health, Henan University of Science and Technology, Luoyang, China; ^4^Animal Diseases and Public Health Engineering Research Center of Henan Province, Luoyang Polytechnic, Luoyang, China

**Keywords:** *Listeria monocytogenes*, glabridin, licorice, subinhibitory concentration, antimicrobial agent

## Abstract

Increases in the virulence and survival of some pathogens in the presence of subinhibitory concentrations of antibiotics have been reported. However, research on the effects of subinhibitory concentrations of antimicrobial substances derived from traditional Chinese medicine on pathogens is still insufficient. Glabridin is a well-known active isoflavone found in licorice roots that possesses a wide range of biological activities. Therefore, in this study, *Listeria monocytogenes* (*L. monocytogenes*) exposed to subinhibitory concentrations of glabridin was used as the research object. The minimum inhibitory concentration (MIC) was determined for *L. monocytogenes*. We investigated the impacts of subinhibitory concentrations of glabridin on the morphology, motility, biofilm formation, adherence, and survival of *L. monocytogenes*. The results indicated that the MIC of glabridin for *L. monocytogenes* was 31.25 μg/mL. At 1/8, 1/4, or 1/2 of the MIC, glabridin did not affect the growth, morphology, flagellar production, or biofilm formation of *L. monocytogenes*. However, subinhibitory concentrations of glabridin inhibited bacterial swimming and swarming motility and decreased the hemolytic activity of *L. monocytogenes*. Glabridin reduced the hemolytic activity of *L. monocytogenes* culture supernatants. The results also showed that subinhibitory concentrations of glabridin had no toxic effect on RAW264.7 cells but decreased the intracellular growth of *L. monocytogenes* in RAW264.7 cells. Furthermore, subinhibitory concentrations of glabridin triggered ROS production but did not induce MET formation in macrophages. In addition, glabridin did not enhance the capacity of *L. monocytogenes* to trigger METs or the extracellular killing of macrophages by METs. Thus, we conclude that subinhibitory concentrations of glabridin reduce *L. monocytogenes* motility and hemolytic activity but do not exhibit antimicrobial activity. Glabridin could be an interesting food additive as a bacteriostatic agent with anti-*Listeria* activity.

## 1 Introduction

*Listeria monocytogenes* (*L. monocytogenes*) is a gram-positive, facultative intracellular bacterial pathogen. It is ubiquitous and widely distributed in soil, vegetation, animal-derived food products, silage, excrement, sewage, and water (Hadjilouka et al., 2016). *L. monocytogenes* is the only *Listeria* species known to be highly pathogenic in humans and is considered to be the most frequently reported foodborne pathogen (Zhang et al., 2024). The occurrence of mild febrile gastroenteritis is one of the most common symptoms in healthy individuals following the consumption of *L. monocytogenes*-contaminated food products. In particular, *L. monocytogenes* leads to more serious invasive diseases, including septicemia, encephalitis, meningitis, peritonitis, recurrent spontaneous abortion (Xu et al., 2024), and even death, in elderly, neonatal, or pregnant patients, as well as chemotherapy-treated patients, transplant recipients, and immunocompromised patients (Budvytyte et al., 2024; Vazquez et al., 2024). The expression of many virulence factors by *L. monocytogenes* potentially contributes to its ability to enter into host cells, survive, and spread to infect adjacent cells (Osek and Wieczorek, 2022). Although large outbreaks of listeriosis have been reported in some countries and regions, these cases account for only a small proportion of all listeriosis cases (Mohapatra et al., 2024), and the case-fatality rate is usually as high as 20–30% in humans (Huang et al., 2023). Approximately 1,600 cases of human listeriosis are reported each year in the United States, 20% of which result in death (Pogreba-Brown et al., 2022). Therefore, *L. monocytogenes* remains a serious public hygiene problem with a heavy economic burden throughout the world, especially in countries with no network management system for complete food hygiene surveillance.

Over the past century, more than 200 antibiotics have been widely used to defend against pathogenic infections and are considered a key therapeutic modality for almost all diseases to date (Mehdi et al., 2018). *L. monocytogenes* is usually susceptible to a wide range of antibiotics in human and veterinary medicine, and the administration of penicillin, ampicillin, and gentamicin is used as the first choice (Arslan and Ozdemir, 2020). The abuse or overuse of antibiotics has led to a rapid increase in the prevalence of antibiotic-resistant bacteria (Mesa-Ramos et al., 2024). Although current antibiotic strategies are still effective options for the treatment of listeriosis (Kumaraswamy et al., 2018), the emergence of multidrug-resistant *L. monocytogenes* isolates from river water, traditionally consumed food products, and human clinical specimens has been documented since the isolation of the first multiresistant strain of *L. monocytogenes* in France in 1988 (Poyart-Salmeron et al., 1990; Harakeh et al., 2009; Soni et al., 2013), and the prevalence rate is increasing in China and other countries (Lachtara et al., 2023; Zhang et al., 2024). Therefore, developing new drugs and alternative intervention approaches for the treatment of listeriosis has become imperative.

Licorice, called Gan-Cao, which has a 2000-year history of application in China, belongs to the genus *Glycyrrhiza* (family: Fabaceae/Leguminosae). It is derived from the roots and stolons of *Glycyrrhiza uralensis Fisch.*, *Glycyrrhiza inflata Bat.*, and *Glycyrrhiza glabra L.* Studies have shown that licorice is used in flavoring and sweetening agents, demulcents, expectorants, and anti-allergic and anti-inflammatory agents (Ji et al., 2024). It has widespread pharmacological effects on human diseases, including atherosclerosis, immunodeficiency, hormone deficiency, endocrine disruption, cancer, and skin diseases (Kwon et al., 2020). The extracts of traditional Chinese herbal medicines contain many effective ingredients. A large number of the active components, including glycyrrhizin acid, glucose, ammonia, polyphenols, flavonoids, isoflavonoids, chalcones, triterpenoids, and saponins, have been isolated from licorice (Pastorino et al., 2018).

Licorice has become the subject of intense global research efforts in recent years (Kuang et al., 2018). At present, these studies have focused mainly on herbal monomers with active components of Chinese herbal extracts. More than 300 flavonoids of licorice, including liquiritin, glycyrrhizic acid, glabridin, liquiritigenin, and isoliquiritigenin, have been identified. Glabridin [(R)-4-(3,4-dihydro-8,8-dimethyl)-2H, 8H-benzo [1,2-b:3,4-b′]dipyran-3yl-1,3-benzenediol, C_20_H_20_O_4_], a polyphenolic flavonoid compound in licorice, is considered one of the key biologically active components. Since the structure of glabridin was first described by Saitoh et al. (1976), it has been found to possess multiple beneficial properties, including antibacterial, antifungal, antiparasitic, antioxidation, anti-inflammatory, antiatherogenic, antifatigue, anticancer, antiobesity, antidiabetes, estrogenic, bone protection, cardiovascular protection, hepatoprotection, and neuroprotective effects (Zhang J. et al., 2023).

Although antibiotics are small molecules with positive and direct therapeutic activity in killing microbes or inhibiting microbial growth, they also have negative effects on bacteria. Exposure to subinhibitory concentrations of antibiotics significantly increases biofilm formation in several pathogenic species (Bernardi et al., 2021). Recently, glabridin was reported to exhibit antimicrobial activity against *L. monocytogenes,* with a minimum inhibitory concentration (MIC) of 12.5 μg/mL (Bombelli et al., 2023). However, little is known about the effects of subinhibitory concentrations of the Chinese herbal medicines on bacteria. Considering the above findings, we were interested in determining whether subinhibitory concentrations of glabridin affect the characteristics of *L. monocytogenes* in this study. For this purpose, we analyzed the effects of subinhibitory concentrations of glabridin on the morphology, motility, biofilm production, adherence, and survival of *L. monocytogenes*.

## 2 Materials and methods

### 2.1 Bacterial strain and growth conditions

The *L. monocytogenes* 10,403 s strain was used in this study and was grown in brain heart infusion (BHI, Haibo, Qingdao, China) broth media supplemented with 1% (w/v) peptone, 1.25% (w/v) dehydrated calf brain infusion, 0.5% (w/v) dehydrated beef heart infusion, 0.5% (w/v) NaCl, 0.2% (w/v) glucose, and 0.25% (w/v) Na_2_PO_4_ under sterile conditions. The medium was filter-sterilized with a 0.2 μm filter (Beyotime Biotechnology, Shanghai, China). Before exposure to glabridin or macrophages, the bacterial cultures were incubated in sterile 50 mL closed conical tubes (Guangzhou Jet Bio-Filtration Co., Ltd., Guangzhou, China) at 37°C under static conditions or with shaking at 180 rpm.

### 2.2 Determination of the MIC and minimum bactericidal concentration (MBC)

Glabridin (molecular weight of 324.40 g/moL) from *Glycyrrhiza glabra L.* root (licorice) was obtained from Luoyang Lansealy Technology Co., Ltd. (Luoyang, China). The antibacterial activity of glabridin against *L. monocytogenes* 10,403 s was determined using broth microdilution methods (MIC and MBC), as previously described (Fang et al., 2018). Briefly, 2-fold serial dilutions of glabridin ranging from 1.95 to 500 μg/mL were prepared and then added to wells containing bacteria (1 × 10^6^ CFUs/ml) in a 96-well plate. The plates were incubated overnight at 37°C. Finally, the MIC was defined as the lowest sample concentration that prevented microbial growth according to the OD_600 nm_. A 1:10 dilution of each concentration in fresh broth was spread onto BHI (Haibo, Qingdao, China) agar plates to determine the MBC. After an incubation at 37°C for 24 h, the MBC was recorded as the minimum concentration at which no apparent growth on agar subculture occurred. Triplicates of this experiment were conducted.

### 2.3 Determination of the growth level

Growth curves were generated to determine the effect of subinhibitory concentrations of glabridin on *L. monocytogenes* growth, as previously described (Jiang et al., 2019). Briefly, cultures of *L. monocytogenes* at the exponential phase in the BHI medium were collected by centrifugation and washed once with fresh BHI medium. Then, the cell’s initial concentration was adjusted to a concentration of 1 × 10^6^ CFUs/ml, and the cells were incubated in BHI media supplemented with subinhibitory concentrations (1/8, 1/4, or 1/2 of the MIC) or the MIC of glabridin with shaking at 37°C. The optical density at 600 nm was measured each hour. Moreover, viable bacteria were counted by serial dilution, plating onto BHI agar, and incubation at 37°C for 24 h.

Moreover, the colony morphology of *L. monocytogenes* was evaluated on BHI agar plates. Cultures of *L. monocytogenes* at the exponential phase in BHI medium were collected by centrifugation and washed once with PBS (10 mM, pH 7.4). Ten microliters (~10^4^ CFUs) of the culture solution were dripped on BHI agar supplemented with subinhibitory concentrations (1/8, 1/4, or 1/2 of the MIC) or the MIC of glabridin. Following an overnight incubation at 37°C, the colony morphology was observed and photographed. The colony growth diameter was measured using a micrometer.

### 2.4 Cellular morphology

Cultures of *L. monocytogenes* at the exponential phase in BHI medium supplemented with subinhibitory concentrations (1/8, 1/4, or 1/2 of the MIC), the MIC, or MBC of glabridin were collected by centrifugation and washed once with PBS (10 mM, pH 7.4) to observe cellular morphology. Then, the cell suspensions were uniformly spread onto sterile glass coverslips. Bacteria were stained with Gram stain and examined under a light microscope.

Moreover, scanning electron microscopy (SEM) was performed via an earlier procedure (Jalil Sarghaleh et al., 2023). Cultures of *L. monocytogenes* at the exponential phase in BHI medium supplemented with subinhibitory concentrations (1/8, 1/4, or 1/2 of the MIC), the MIC, or MBC of glabridin were collected by centrifugation and washed once with PBS (10 mM, pH 7.4). Then, the cell suspensions were uniformly spread onto sterile glass coverslips. A 2.5% (v/v) glutaraldehyde solution (Servicebio, Wuhan, China) was used for stabilization and incubated with the samples at 4°C for 2 h. After washing with distilled water, ethanol gradients (25, 50, 75, 95, and 100%) were used for the final dehydration. After air drying at room temperature, critical point drying was performed with liquid CO_2_. The samples were covered with a layer of gold and then observed under a JEOL scanning electron microscope (JSM-IT200 InTouchScope™, JEOL, Tokyo, Japan).

### 2.5 Motility assay

The motility phenotype was assayed on soft BHI agar as described previously (Roy et al., 2022), with minor modifications. Briefly, 0.25% (w/v) and 0.5% (w/v) agar were mixed with BHI for the swimming and swarming studies, respectively. Cultures (~10^4^ CFUs) at the exponential phase were point inoculated onto BHI agar plates supplemented with subinhibitory concentrations (1/8, 1/4, or 1/2 of the MIC) of glabridin. The culture medium was incubated at 30°C for 24 h, and the migration zone (mm) was determined to report the bacterial motility in the agar by measuring the diameter using a micrometer. Each group was independently tested three times.

### 2.6 Transmission electron microscopy (TEM)

TEM experiments were performed to investigate the flagella presence as described previously (Wang et al., 2024). Bacteria from 0.25% BHI agar plates supplemented with subinhibitory concentrations (1/8, 1/4, or 1/2 of the MIC) of glabridin were fixed with a 3% paraformaldehyde solution on an electron microscopy grid. Then, the grids were washed three times. The bacteria were stained with phosphotungstic acid (2%) for 10 s at room temperature, the excess liquid was gently removed using filter paper, and the grids were air-dried. The dried grids were observed under a Hitachi transmission electron microscope (HT7700, Hitachi High-Tech Corporation, Tokyo, Japan).

### 2.7 Biofilm formation assays

The effect of subinhibitory concentrations of glabridin on the formation of biofilms was determined using a crystal violet assay according to the procedure described by Djordjevic et al. (2002), with slight modifications. Assays were performed in sterile 96-well plates. Briefly, overnight cultures were added to the wells with 100 μL of fresh medium supplemented with subinhibitory concentrations (1/8, 1/4, or 1/2 of the MIC), the MIC or MBC of glabridin. The plates were placed in an incubator at 37°C for 48 h. Subsequently, the supernatant was discarded, and then the wells were gently washed three times with aseptic PBS. The plates were blotted dry on a sterilized paper towel after the last wash and allowed to dry naturally for 1 h. Then, the biofilms were stained with 2% (m/v) crystal violet for 45 min. The wells were washed with distilled water to remove the unbound dye. After drying at 37°C for 10 min, the cell-bound dye was dissolved in 200 μL of 95% (w/w) ethanol. The quantitative evaluation of biofilm formation was performed on a microplate reader (Infinite M Nano, TECAN, Switzerland) by measuring the absorbance of the solution at 570 nm.

Moreover, the biofilm formation of *L. monocytogenes* was investigated using confocal laser scanning microscopy (CLSM) (Adamus-Bialek et al., 2015). Initially, overnight cultures were seeded onto 20-mm glass cover slips in 12-well plates in the absence or presence of glabridin. After an incubation at 37°C for 48 h to allow biofilm formation, the culture medium was discarded, and then the wells were gently washed three times with aseptic PBS. Subsequently, the biofilms were stained with SYTO Green (KeyGEN BioTECH, Nanjing, China) and PI (Beyotime Biotechnology, Shanghai, China), a green and red fluorescent nucleic acid stain, for up to 20 min at room temperature in the dark. The samples were visualized under a confocal laser scanning microscope (FV3000, Olympus, Japan) at an excitation wavelength of 500 nm and emission wavelength of 535 nm, according to the manufacturer’s instructions.

SEM was used to visualize the morphologies of the *L. monocytogenes* biofilms, as previously described (Wang et al., 2022), with slight modifications. The fresh cultures were placed on 20-mm glass cover slips in the wells of 12-well plates. After the incubation, the attached cells were washed three times with PBS to remove the planktonic cells. The samples were fixed with a 2.5% (v/v) glutaraldehyde solution (Servicebio, Wuhan, China) at 4°C for 10 h. After washes with distilled water, the samples were subjected to dehydration with an increasing series of ethanol solutions (25, 50, 75, 95, and 100%). After air drying at room temperature, critical point drying was performed with liquid CO_2_. Subsequently, the samples were covered with a layer of gold using a sputter coater and examined using a JEOL scanning electron microscope (JSM-IT200 InTouchScope^™^, JEOL, Tokyo, Japan).

### 2.8 Biofilm eradication assays

The evaluation of the biofilm-eradicating ability of glabridin was performed according to the previously described methods with further modifications (Shen et al., 2021). Briefly, overnight cultures were added to 96-well plates and incubated for 48 h at 37°C to form a biofilm. After the incubation, the culture medium was removed and replaced with sterile BHI medium by washing three times with PBS. Subsequently, subinhibitory concentrations (1/8, 1/4, or 1/2 of the MIC), the MIC, and MBC of glabridin were added to the wells. The plates were incubated at 37°C for 1 h. After the incubation, the methods mentioned above (crystal violet assay, CLSM, and, SEM) were used to evaluate biofilm eradication.

### 2.9 Hemolytic activity assay

Hemolytic activity was assayed using the blood-plate method as previously described (Kawacka et al., 2022). Briefly, 5.0% of sterile sheep blood was mixed with BHI to prepare a blood agar plate. *L. monocytogenes* cultures with logarithmic growth (~10^4^ CFUs) at the exponential phase were point-inoculated onto BHI agar plates supplemented with subinhibitory concentrations (1/8, 1/4, and 1/2 of MIC) of glabridin. The culture medium was incubated at 37°C for 24 h. The plates were then visually examined for clear zones or no zones around colonies. The experiments were independently repeated three times.

Moreover, the hemolytic activity was determined by performing a microdilution method according to a previous study (Yue et al., 2023). Briefly, *L. monocytogenes* cultures with logarithmic growth (~10^6^ CFUs) were treated with subinhibitory concentrations (1/8, 1/4, or 1/2 of the MIC) of glabridin at 37°C and 120 rpm for 12 h. Bacterial cultures were centrifuged at 12,000 rpm for 10 min. Then, the supernatant was filtered through a 0.22 μm pore filter (Guangzhou Jet Bio-Filtration Co., Ltd., Guangzhou, China). After filtration, 100 μL of the cell-free supernatant was mixed with 100 μL of a 5.0% (v/v) red blood cell suspension in a 96-well plate and incubated for 1 h at 37°C. Subsequently, the tested samples were gathered by centrifugation at 1000 rpm for 5 min. Finally, absorbance measurements were performed at 540 nm with a microplate reader. BHI and 1% Triton X-100 were served as negative and positive controls, respectively. The percentage of hemolysis (%) = (OD_540_ nm of test well – OD_540_ nm of negative control)/(OD_540_ nm of positive control – OD_540_ nm of negative control) × 100%.

### 2.10 Real-time PCR

*L. monocytogenes* cultures with logarithmic growth were initially treated with subinhibitory concentrations (1/8, 1/4, or 1/2 of the MIC) of glabridin at 37°C and 120 rpm for 12 h. The samples were centrifuged at 12,000 rpm for 5 min, and the supernatant was discarded. Total RNA was extracted using a TRNpure Total RNA Kit (Nobelab Biotech, Beijing, China) based on the manufacturer’s manual. The RNA concentration, purity, and integrity were assessed using a NanoDrop 2000c (Thermo, Massachusetts, United States). Then, the cDNA strand was synthesized using an EasyScript^®^ All-in-One First-Strand cDNA Synthesis SuperMix for qPCR (One-Step gDNA Removal) (TransGen, Beijing, China), according to the manufacturer’s protocol. Reactions (20 μL) containing 100 ng of RNA, the mix, and primers were prepared. The thermal cycling conditions were 42°C for 15 min and 85°C for 5 s using a T100 Thermal Cycler (T100™; Bio-Rad Laboratories, California, United States). cDNA was quantified using a NanoDrop 2000c. Beacon Designer (version 8.0) software was used to design specific primers for the *hly* (GenBank: DQ054589.1; forward: 5′-TGCAAGTCCTAAGACGCCA-3′, reverse: 5′-CACTGCATCTCCGTGGTATACTAA-3′) gene (Tamburro et al., 2015). The *16S rRNA* (GenBank: CP002002.1; forward: 5′-TTAGCTAGTTGGTAGGGT-3′, reverse: 5′-AATCCGGACAACGCTTGC-3′) gene is considered a housekeeping gene for *L. monocytogenes* (Fraser et al., 2003). The amplification profile was as follows: predenaturation at 94°C for 30 s and 40 cycles of 5 s at 94°C and 30 s at 60°C. Reactions (20 μL) containing PerfectStart^®^ Green qPCR SuperMix (TransGen, Beijing, China), cDNA, and specific primers were performed in a CFX96 real-time system (Bio-Rad Laboratories, California, United States). All samples were prepared in triplicate. The mRNA expression level of the *hly* gene was normalized to the expression level of *16S rRNA.* The expression levels in the untreated group were normalized to 1 for comparison with those in the treated group. The relative expression levels of the *hly* gene were analyzed using the 2^−ΔΔCT^ relative expression quantification method: Δ^ΔCt^ = (mean Ct value of *hly* in the sample – mean Ct value of *16S rRNA* in the sample) – (mean Ct value of *hly* in the control – mean Ct value of *16S rRNA* in the control).

### 2.11 Eukaryotic cell line and culture conditions

RAW264.7 cells were purchased from Procell Life Science and Technology Co., Ltd. (Wuhan, China). The RAW264.7 cells and Caco-2 cells were stored in the Luoyang Key Laboratory of Live Carrier Biomaterial and Animal Disease Prevention and Control. The cells were grown in DMEM (HyClone, United States) supplemented with 10% heat-inactivated (56°C, 30 min) fetal bovine serum (FBS; Clark Bioscience, Dalian, United States) and maintained at 37°C with 5% CO_2_ in a humidified incubator. Then, 100 U/mL penicillin and 100 μg/mL streptomycin (Beijing Solarbio Science & Technology Co., Ltd., Beijing, China) were added when needed. The cells were passaged in 25 cm^2^ flasks (Guangzhou Jet Bio-Filtration Co., Ltd., Guangzhou, China). After the cells were counted using a hemocytometer, they were seeded into 24-well plates (Guangzhou Jet Bio-Filtration Co., Ltd., Guangzhou, China) for subsequent procedures.

### 2.12 Cytotoxicity test

The cytotoxicity of glabridin was evaluated using a lactate dehydrogenase (LDH) assay kit as previously described (Hou et al., 2023). Briefly, RAW264.7 cells and Caco-2 cells were seeded at a density of 2 × 10^5^ cells per well in a 24-well cell culture plate, respectively. The cells were incubated in DMEM supplemented with subinhibitory concentrations (1/8, 1/4, or 1/2 of the MIC), the MIC, or MBC of glabridin for 6 h. The cells treated with 0.2% Triton X-100 or DMEM only were used as the positive and negative controls, respectively. The cell culture supernatants were centrifuged and collected. The levels of LDH release were measured using an LDH Cytotoxicity Assay Kit (Beyotime Biotechnology, Shanghai, China) in accordance with the manufacturer’s instructions. The absorbance at 490 nm was read using a microplate reader. The results are shown as (OD_490_ nm of sample – OD_490_ nm of negative control)/(OD_490_ nm of positive control – OD_490_ nm of negative control) × 100%.

### 2.13 Intracellular growth assay in macrophages

The intracellular growth assay was performed as previously described (Meng et al., 2015; Zhou et al., 2017). RAW264.7 cells were seeded at a density of 2 × 10^5^ cells per well in 24-well plates with antibiotic-free medium. The cells were incubated with subinhibitory concentrations (1/8, 1/4, or 1/2 of the MIC) of glabridin, infected with bacteria at an MOI of 10:1, and then incubated at 37°C with 5% CO_2_ for 1 h. The cells were rinsed three times with PBS and incubated with 100 μg/mL gentamicin for 1 h to kill the extracellular bacteria. Then, the cells were washed three times with PBS to remove the killed extracellular bacteria. The infected RAW264.7 cells were then incubated with fresh DMEM supplemented with subinhibitory concentrations (1/8, 1/4, or 1/2 of the MIC) of glabridin and 10% FBS for 3h. The cells were subsequently lysed with 0.1% Triton X-100 in PBS for 5 min. The number of viable intracellular bacteria was determined by plating serial dilutions of the resulting lysates on BHI agar plates. The experiments were repeated in three independent assays.

### 2.14 MET formation in macrophages

Macrophage extracellular traps (METs) were visualized as previously described (Cogen et al., 2010; Brinkmann et al., 2012). RAW264.7 cells (2 × 10^5^ cells per well) were plated onto laser confocal Petri dishes in DMEM without antibiotics or fetal calf serum. The cells were incubated with subinhibitory concentrations (1/8, 1/4, or 1/2 of the MIC) of glabridin, infected with *L. monocytogenes* at an MOI of 10:1, and then incubated at 37°C with 5% CO_2_ for 2 h. The cells were stained with Hoechst 33342 (Beyotime Biotechnology, Shanghai, China) and observed via confocal laser scanning microscopy (FV3000, Olympus, Japan) at 350 nm excitation and 460 nm emission. Images of random fields were acquired from each group (Zhang X. W. et al., 2023). The MET formation in the PMA-treated macrophages was set as 100%. The data are presented as percentages of the PMA-treated group.

### 2.15 Intracellular ROS generation

Intracellular ROS generation was detected according to a previously described procedure (Liu et al., 2014). RAW264.7 cells (2 × 10^5^ cells per well) were seeded onto laser confocal Petri dishes in DMEM without antibiotics or fetal calf serum. The cells were incubated with subinhibitory concentrations (1/8, 1/4, or 1/2 of the MIC) of glabridin, infected with *L. monocytogenes* at an MOI of 10:1, and then incubated at 37°C with 5% CO_2_ for 2 h. DCFH-DA (5 μM, Beyotime, Shanghai, China) was added, and the cells were continuously incubated in the dark. The samples were observed under a confocal laser scanning microscope at 488 nm excitation and 525 nm emission.

### 2.16 MET-mediated extracellular killing assay in macrophages

The MET-mediated extracellular killing assay was performed as described previously (Liu et al., 2014). Briefly, 2 × 10^5^ RAW264.7 cells per well were seeded into a 24-well plate. The cells were incubated with subinhibitory concentrations (1/8, 1/4, or 1/2 of the MIC) of glabridin, infected with *L. monocytogenes* at an MOI of 10:1, and then incubated at 37°C with 5% CO_2_ for 2 h. Cytochalasin D (10 μg/mL; Beijing Solarbio Science & Technology Co., Ltd., Beijing, China) or 100 U/mL protease-free DNase I (Sangon Biotech (Shanghai) Co., Ltd., Shanghai, China) was added to inhibit phagocytosis or MET formation at 20 min before infection with *L. monocytogenes*. Pretreatment with cytochalasin D and DNase I was used as the 100% survival control group. The experiments were repeated five times. Bacteria were serially diluted and evenly plated onto BHI agar to determine the number of CFUs.

### 2.17 Statistical analysis

All experiments were performed in three independent replicates. The data are presented as means ± SEMs. Statistical significance was determined using two-tailed Student’s *t*-test. *p* values indicated in the figures (*p < 0.05 and **p < 0.01) were considered significant.

## 3 Results

### 3.1 Effect of subinhibitory concentrations of glabridin on the growth of *L. monocytogenes*

**Figure 1:**
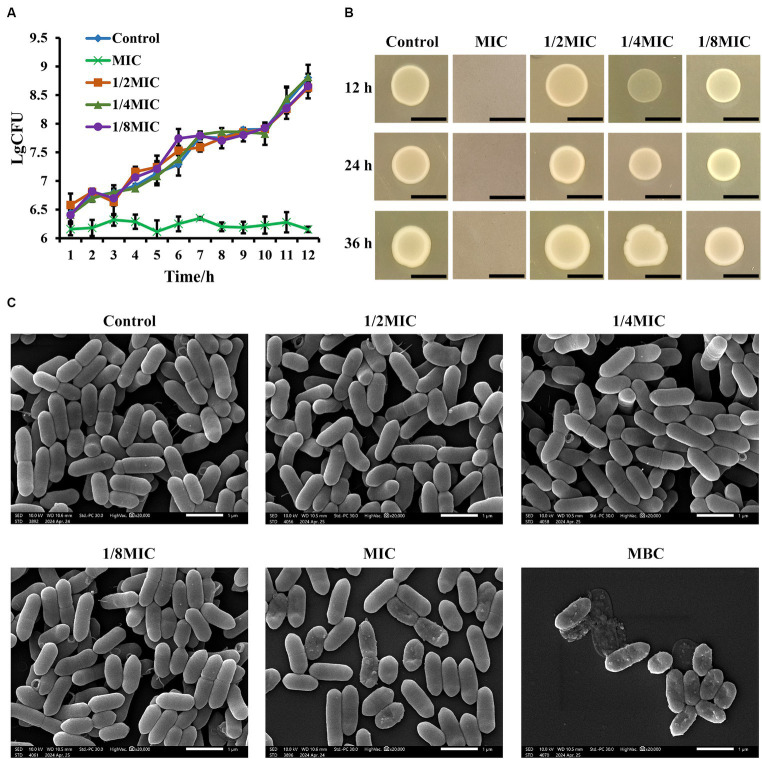
Effect of subinhibitory concentrations of glabridin on the growth and morphology of *L. monocytogenes.* **(A)** Growth curves of *L. monocytogenes* in subinhibitory concentrations (1/8, 1/4, or 1/2 of the MIC) or the MIC of glabridin. There was no statistically significant difference between bacterial growth of the glabridin-treated and -untreated cultures after overnight incubation in BHI medium. **(B)** Colony morphology of *L. monocytogenes* in subinhibitory concentrations of glabridin. *L. monocytogenes* treated with subinhibitory concentrations (1/8, 1/4, or 1/2 of the MIC) or the MIC of glabridin were grown in BHI agar, and no difference in colony morphology was observed. Scale bar: 5 mm. **(C)** Scanning electron microscopy images of the cellular morphology of *L. monocytogenes* treated with subinhibitory concentrations (1/8, 1/4, or 1/2 of the MIC), the MIC or MBC of glabridin. *L. monocytogenes* were grown in BHI medium and observed under a Hitachi scanning electron microscope. Scale bar: 1 μm. The experiments were repeated three times.

As determined using the microdilution method, the MIC value for *L. monocytogenes* was 31.25 μg/mL of glabridin. The results indicated that the MBC of glabridin against *L. monocytogenes* was 125 μg/mL. The optical densities of the bacterial suspension were measured at 600 nm to determine the effect of subinhibitory concentrations of glabridin on the growth of *L. monocytogenes*. Following an overnight incubation in BHI medium, the growth profiles of glabridin-treated and -untreated *L. monocytogenes* were similar (Supplementary Figure S1A). As shown in Figure 1, the growth of *L. monocytogenes* was not significantly affected by glabridin concentrations of 3.91, 7.81, or 15.63 μg/mL, which are equal to 1/8, 1/4, or 1/2 of the MIC of glabridin, respectively (Figure 1A). Moreover, the colony morphologies of the glabridin-treated and -untreated *L. monocytogenes* strains were compared. The detailed results are presented in Figure 1B. The colony morphology of the glabridin-treated *L. monocytogenes* after incubation with subinhibitory concentrations was similar to that of the glabridin-untreated *L. monocytogenes* in BHI agar plates (Figure 1B). As the glabridin concentration increased, no significant differences in colony size were observed (Supplementary Figure S1B). The colonies of all groups appeared as milky white, opaque colonies with smooth surfaces. Therefore, our results suggested that subinhibitory concentrations of glabridin did not affect the growth of *L. monocytogenes*.

### 3.2 Effect of subinhibitory concentrations of glabridin on the morphology of *L. monocytogenes*

Gram staining and SEM were performed to determine the effect of subinhibitory concentrations of glabridin on the morphology of *L. monocytogenes*. The light microscopic examination of bacterial staining indicated that *L. monocytogenes* treated with 1/8, 1/4, or 1/2 of the MIC for glabridin had the same cellular morphology as the glabridin-untreated *L. monocytogenes* (Supplementary Figure S1C). The microscopic appearance of the glabridin-treated *L. monocytogenes* was further observed via SEM. SEM images of *L. monocytogenes* before and after treatment with subinhibitory concentrations of glabridin (1/8, 1/4, or 1/2 of the MIC) are shown in Figure 1C. The structure of *L. monocytogenes* is naturally rod-shaped. Treatment of the bacteria with glabridin (1/8, 1/4, or 1/2 of the MIC) did not cause changes in the surface morphology (Figure 1C). Under a light microscope, the results revealed that the number of bacteria in the group treated with the MIC of glabridin was significantly lower than the number of bacteria in the groups treated with subinhibitory concentrations of glabridin (Supplementary Figure S1C). However, SEM confirms the shrunken, crumpled appearance of the membranes of some bacteria in the group treated with the MIC of glabridin (Figure 1C). Moreover, the bacteria were disrupted after the application of the MBC of glabridin (Figure 1C). Therefore, in the following experiments in this study, subinhibitory concentrations of glabridin, 1/8, 1/4, or 1/2 of the MIC, were used since they did not affect the growth or morphology of *L. monocytogenes*.

### 3.3 Subinhibitory concentrations of glabridin inhibit the motility of *L. monocytogenes*

**Figure 2:**
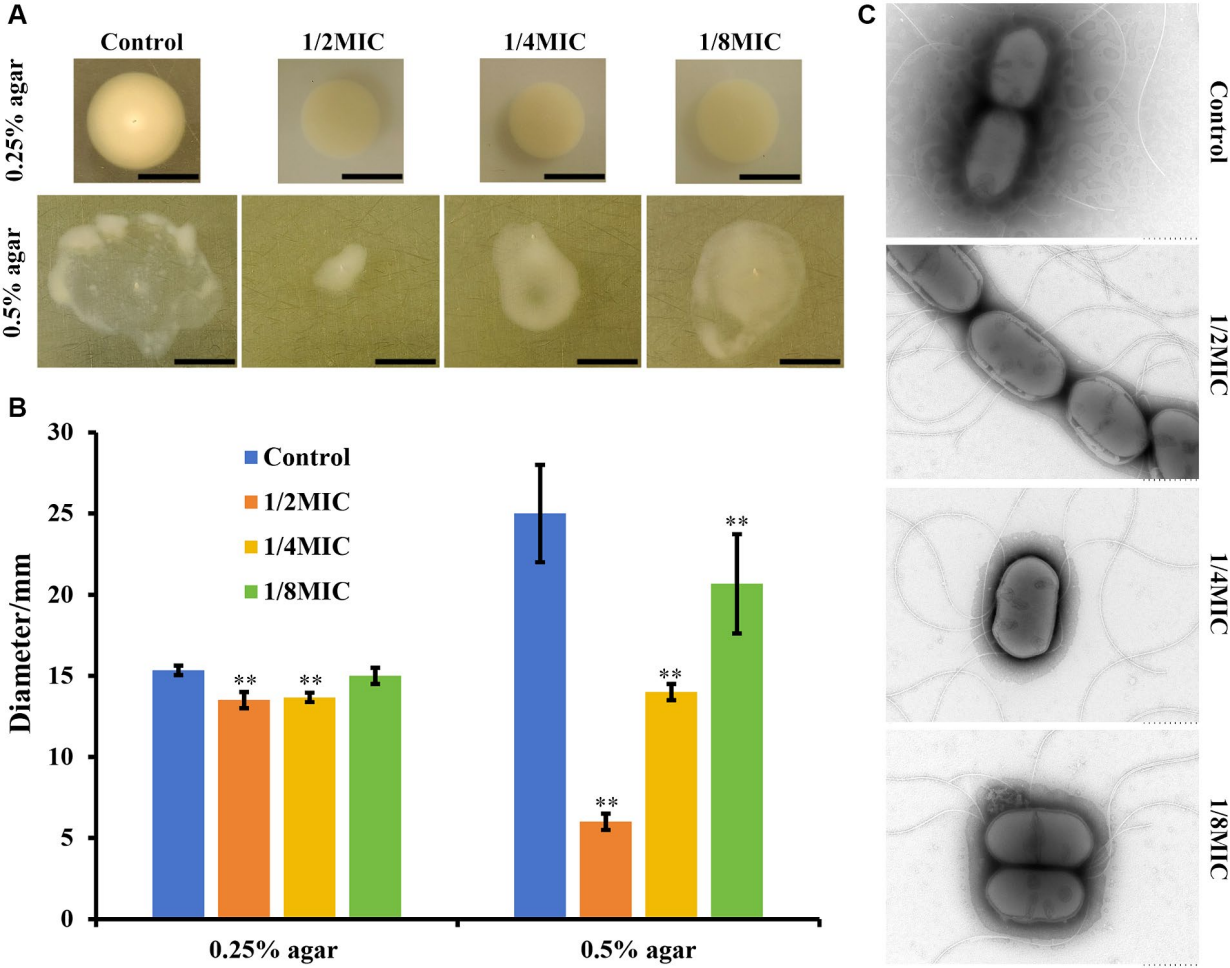
Motility of *L. monocytogenes* in subinhibitory concentrations (1/8, 1/4, or 1/2 of the MIC) of glabridin in semisoft agar plates. **(A)** The bacterial cultures (~10^4^ CFUs) at the exponential phase were spotted on the center of freshly prepared BHI with 0.3% (w/v) and 0.5% (w/v) agar for the swimming and swarming studies, respectively. Subinhibitory concentrations (1/8, 1/4, or 1/2 of the MIC) of glabridin were added. After an incubation for 24 h at 30°C, the plates were imaged and measured using calipers. **(B)** Representative images and the diameter of swimming and swarming zones. ^**^p < 0.01 indicates statistical significance compared with the control. **(C)** Transmission electron microscopic visualization of the effects of glabridin on flagella production. *L. monocytogenes* was grown in 0.3% BHI agar plates in the absence or presence of subinhibitory concentrations (1/8, 1/4, or 1/2 of the MIC) of glabridin. Bacteria were visualized using transmission electron microscopy with negative staining. Scale bar: 500 nm.

Flagellum-mediated motility is involved in the bacterial pathogenicity, biofilm formation, drug resistance, and chronic infections. Thus, we examined whether subinhibitory concentrations of glabridin affect the motility of *L. monocytogenes in vitro*. A semisoft agar assay with 0.3% (w/v) and 0.5% (w/v) agar was used to monitor the swimming and swarming of *L. monocytogenes*, respectively. In the swimming test, we observed a significant decrease in the mobility of *L. monocytogenes* exposed to glabridin (1/4 or 1/2 of the MIC) through soft agar (Figure 2A). In addition, the quantitative experimental results showed that the swimming expansion rate of *L. monocytogenes* colonies in the presence of 1/8 of the MIC of glabridin did not differ from that of glabridin-untreated *L. monocytogenes* colonies (Figure 2B). As depicted in Figure 2, for the swarming test, the motility of *L. monocytogenes* decreased with increasing concentrations of glabridin (1/8, 1/4, or 1/2 of the MIC) in 0.5% BHI agar, and the effect was concentration-dependent (Figure 2A). The zone size of *L. monocytogenes* exposed to glabridin at a concentration of 1/2 of the MIC was significantly lower than that at a concentration of 1/8 of the MIC (Figure 2B). Therefore, subinhibitory concentrations of glabridin inhibited bacterial swimming and swarming motility in *L. monocytogenes*.

### 3.4 Effect of subinhibitory concentrations of glabridin on flagellar production in *L. monocytogenes*

TEM was used to further determine whether subinhibitory concentrations of glabridin adversely affected flagella synthesis. Interestingly, the results showed that *L. monocytogenes* exposed to different concentrations of glabridin were able to produce flagellar filaments, as shown by negative staining of the glabridin-untreated *L. monocytogenes* (Figure 2C). No significant difference was found. Therefore, the results clearly showed that subinhibitory concentrations of glabridin did not interfere with flagellar production in *L. monocytogenes*.

### 3.5 Effect of subinhibitory concentrations of glabridin on *L. monocytogenes* biofilms

**Figure 3:**
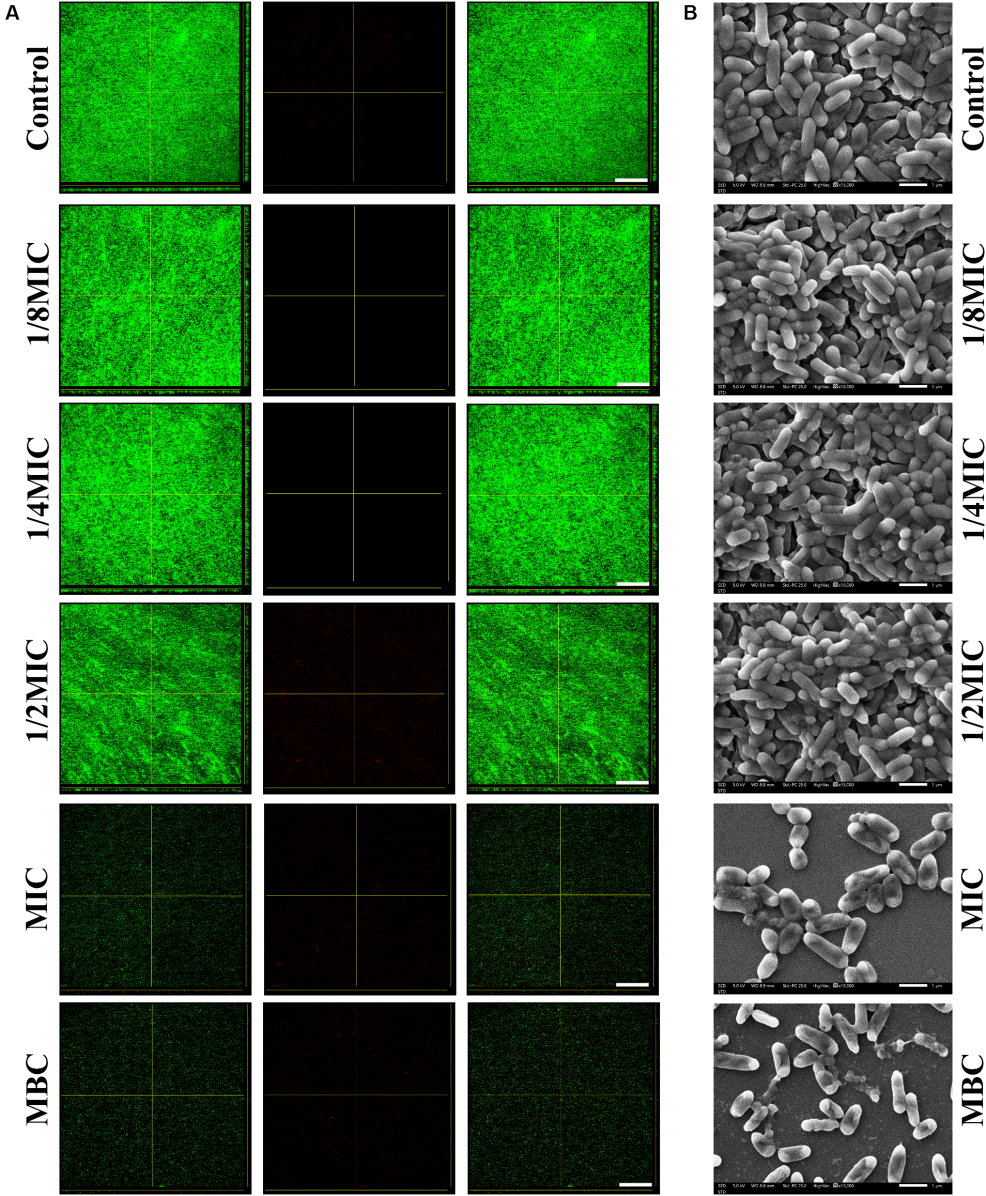
Biofilm formation of *L. monocytogenes* in the presence of subinhibitory concentrations (1/8, 1/4, or 1/2 of the MIC) of glabridin. **(A)** Confocal laser scanning microscopy images of biofilms of *L. monocytogenes* treated with subinhibitory concentrations (1/8, 1/4, or 1/2 of the MIC), the MIC or MBC of glabridin after 48 h of incubation at 37°C. Scale bar: 200 μm. **(B)** Scanning electron microscopy images of biofilms of *L. monocytogenes* treated with subinhibitory concentrations (1/8, 1/4, or 1/2 of the MIC), the MIC or MBC of glabridin after 48 h of incubation at 37°C. Scale bar: 1 μm.

Bacteria were grown on polystyrene in BHI plates supplemented with subinhibitory concentrations (1/8, 1/4, or 1/2 of the MIC) of glabridin to study whether subinhibitory concentrations of glabridin influence biofilm formation of *L. monocytogene in vitro*. Biofilm formation was quantified after 48 h by performing crystal violet staining. As shown in Figure 3A, *L. monocytogenes* produced a biofilm in the absence of glabridin. Although a declining trend in biomass was observed after the bacteria were exposed to subinhibitory concentrations of glabridin, a statistically significant difference was not observed after culture in the absence or presence of glabridin (Supplementary Figure S2A). The corresponding fluorescence microscopy images are shown in Figure 3A. Consistent with the above results, *L. monocytogenes* showed a similar phenomenon in terms of the biofilm biomass with or without glabridin treatment (Figure 3A). Since a green color is used to stain cells, the cells treated with or without glabridin were clearly green and observed in the form of dots. Moreover, very small amounts of biofilm production were observed after treatment with glabridin at the MIC but not at the MBC (Figure 3A). SEM imaging revealed biofilm morphologies and supported the CLSM results. Based on the SEM images, many microcolonies that held together were observed in the absence of glabridin, whereas no change occurred after treatment with subinhibitory concentrations (1/8, 1/4, or 1/2 of the MIC) of glabridin (Figure 3B). However, compared with those in the control group, a few cells were attached to the glass coupons after treatment with glabridin at the MIC, and the bacteria exhibited a normal morphology. Interestingly, the bacteria were disrupted after the application of glabridin at the MBC (Figure 3B), and the bacterial morphology was obviously wrinkled.

**Figure 4:**
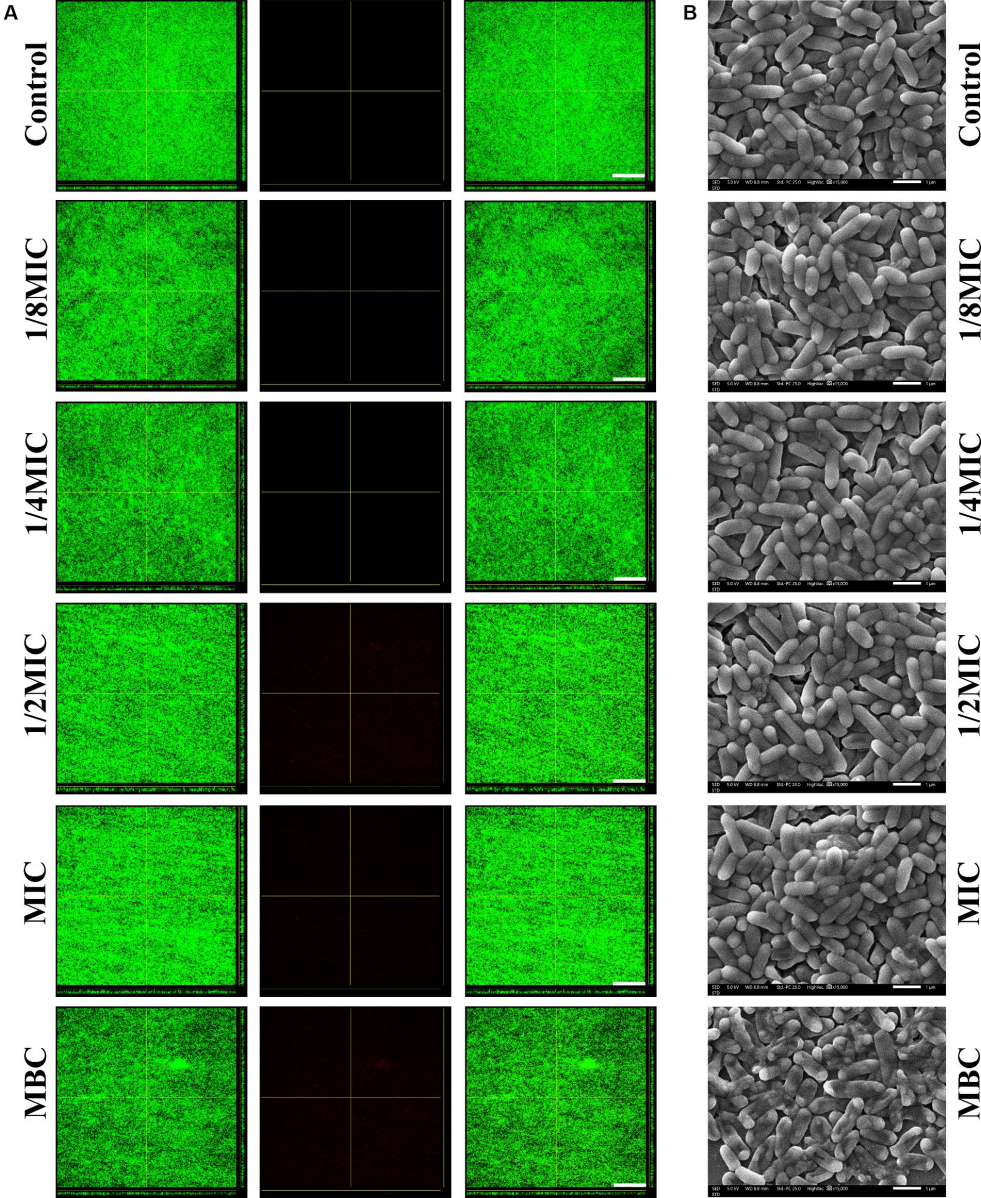
Eradication of *L. monocytogenes* mature biofilms in the presence of subinhibitory concentrations (1/8, 1/4, or 1/2 of the MIC) of glabridin. **(A)** Confocal laser scanning microscopy examination for eradication of *L. monocytogenes* mature biofilms by glabridin. The 48 h mature biofilms on cover slide were cultured with or without subinhibitory concentrations (1/8, 1/4, or 1/2 of the MIC), the MIC or MBC of glabridin, then examined by confocal laser scanning microscopy. Scale bar: 200 μm. **(B)** Scanning electron microscopy examination for eradication of *L. monocytogenes* mature biofilms by glabridin. The 48 h mature biofilms on cover slide were cultured with or without subinhibitory concentrations (1/8, 1/4, or 1/2 of the MIC), the MIC or MBC of glabridin, then fixed and examined by scanning electron microscopy. Scale bar: 1 μm.

According to the crystal violet staining results, none of the subinhibitory concentrations (1/8, 1/4, or 1/2 of the MIC) or the MIC of glabridin could eradicate the complete biofilms of *L. monocytogenes* (Figure 4A; Supplementary Figure S2B). What is more, at the MBC, glabridin did not eradicate the formed biofilms. Furthermore, CLSM images indicated that subinhibitory concentrations (1/8, 1/4, or 1/2 of the MIC) or the MIC of glabridin had no obvious effect on the biofilms (Figure 4B). Then, SEM images of the bacterial biofilms were recorded. The thick and dense layer of bacteria in the control group was smooth and intact (Figure 4B), similar to that observed after treatment with subinhibitory concentrations (1/8, 1/4, or 1/2 of the MIC) or the MIC of glabridin (Figure 4B). When the bacteria were exposed to glabridin at the MBC, the bacteria exhibited obvious wrinkling (Figure 4B). These results suggested that subinhibitory concentrations of glabridin did not alter *L. monocytogenes* biofilms.

### 3.6 Subinhibitory concentrations of glabridin decrease the hemolytic activity of *L. monocytogenes*

**Figure 5:**
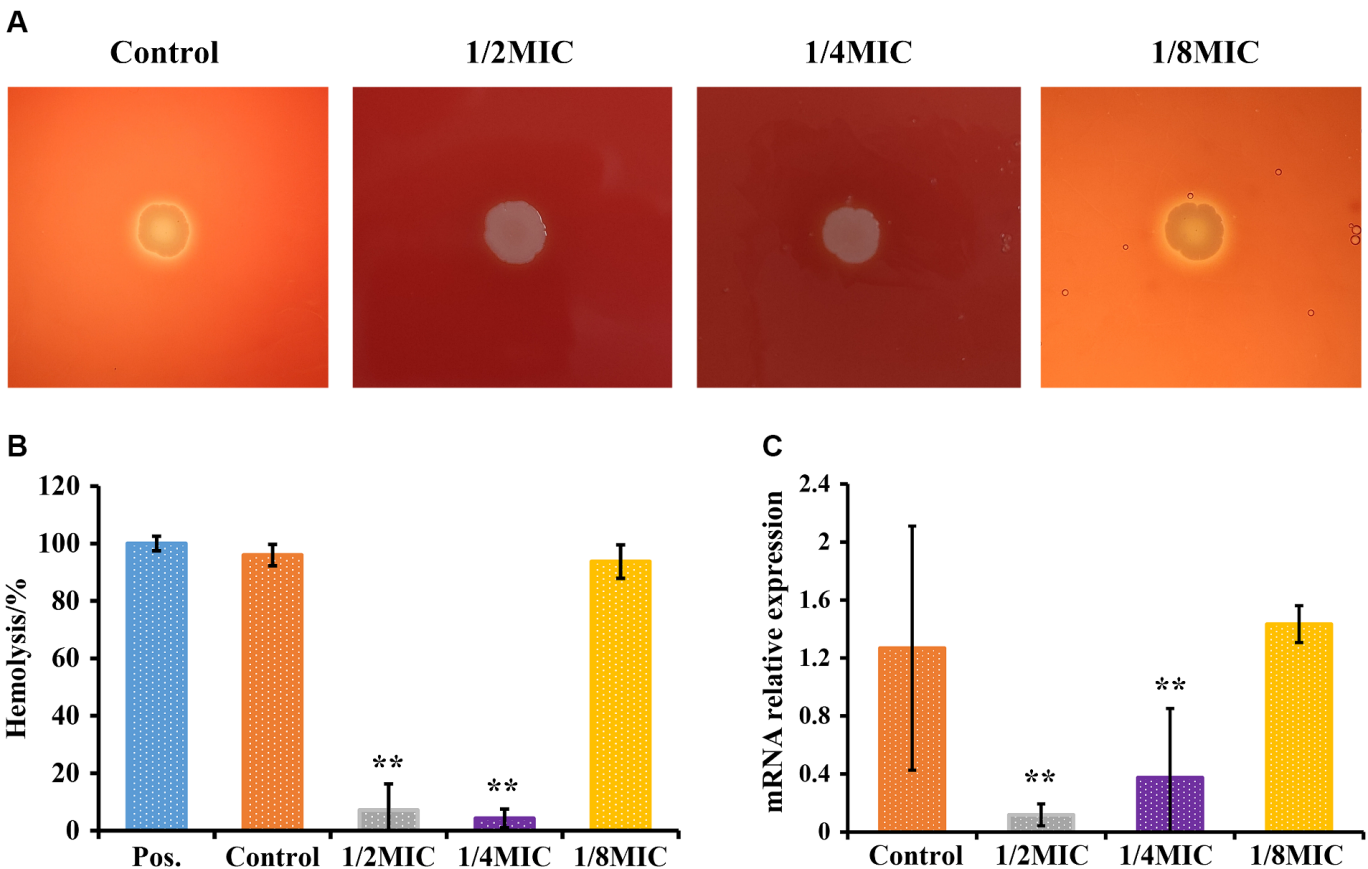
Effect of glabridin at subinhibitory concentrations on the hemolytic activity of *L. monocytogenes*. **(A)** The hemolytic activity of *L. monocytogenes* was analyzed using blood agar plates. *L. monocytogenes* was point inoculated onto BHI agar plates supplemented with 5.0% of sterile sheep blood and subinhibitory concentrations (1/8, 1/4, or 1/2 of the MIC) of glabridin. **(B)** The hemolytic activity of *L. monocytogenes* was analyzed by performing a microdilution method. *L. monocytogenes* were cultured at 37°C for 12 h exposure to subinhibitory concentrations (1/8, 1/4, or 1/2 of the MIC) of glabridin. Filtered supernatants were added at a 1:1 ratio to a suspension of red blood cells, followed by incubation for 1 h at 37°C. The optical density at 540 nm was measured. Triton X-100 was used as the positive control (Pos.). **(C)** Expression level of the *hly* gene in *L. monocytogenes* by RT-PCR. Each group was independently repeated three times. *L. monocytogenes* were cultured and exposed to subinhibitory concentrations (1/8, 1/4, or 1/2 of the MIC) of glabridin at 12 h of incubation at 37°C. mRNA was extracted. The abundances of the *hly* transcripts were quantified. Data were normalized to the *16S rRNA* levels. The experiments were repeated three times. Statistical analysis was carried out using a Student’s *t*-test. ^**^p < 0.01 (*p* < 0.05 or less was regarded as statistically significant).

A bacterial plate assay was used to determine hemolytic activity. As shown in Figure 5A, *L. monocytogenes* 10403S without glabridin treatment exhibited hemolytic activity on blood agar plates. No difference in the hemolytic ability was observed between untreated *L. monocytogenes* and *L. monocytogenes* treated with glabridin at 1/8 of the MIC. However, none of the *L. monocytogenes* strains cultured in the presence of glabridin at 1/4 or 1/2 of the MIC showed hemolytic activity (Figure 5A). The microdilution method also indicated that the addition of glabridin at 1/4 or 1/2 of the MIC resulted in a dramatic decrease in hemolytic activity (Figure 5B). Moreover, the expression of the *hly* gene was significantly higher in *L. monocytogenes* treated with glabridin at 1/4 or 1/2 of the MIC than in untreated *L. monocytogenes*. The expression of the *hly* gene in *L. monocytogenes* treated with glabridin at 1/4 or 1/2 of the MIC decreased significantly by more than 5-fold compared with that in *L. monocytogenes* treated with glabridin at 1/8 of the MIC (*p* < 0.001), as shown in Figure 5C. Therefore, these results clearly showed that subinhibitory concentrations of glabridin decreased the hemolytic activity of *L. monocytogenes*.

### 3.7 Subinhibitory concentrations of glabridin are not toxic to Caco-2 and RAW264.7 cells

**Figure 6:**
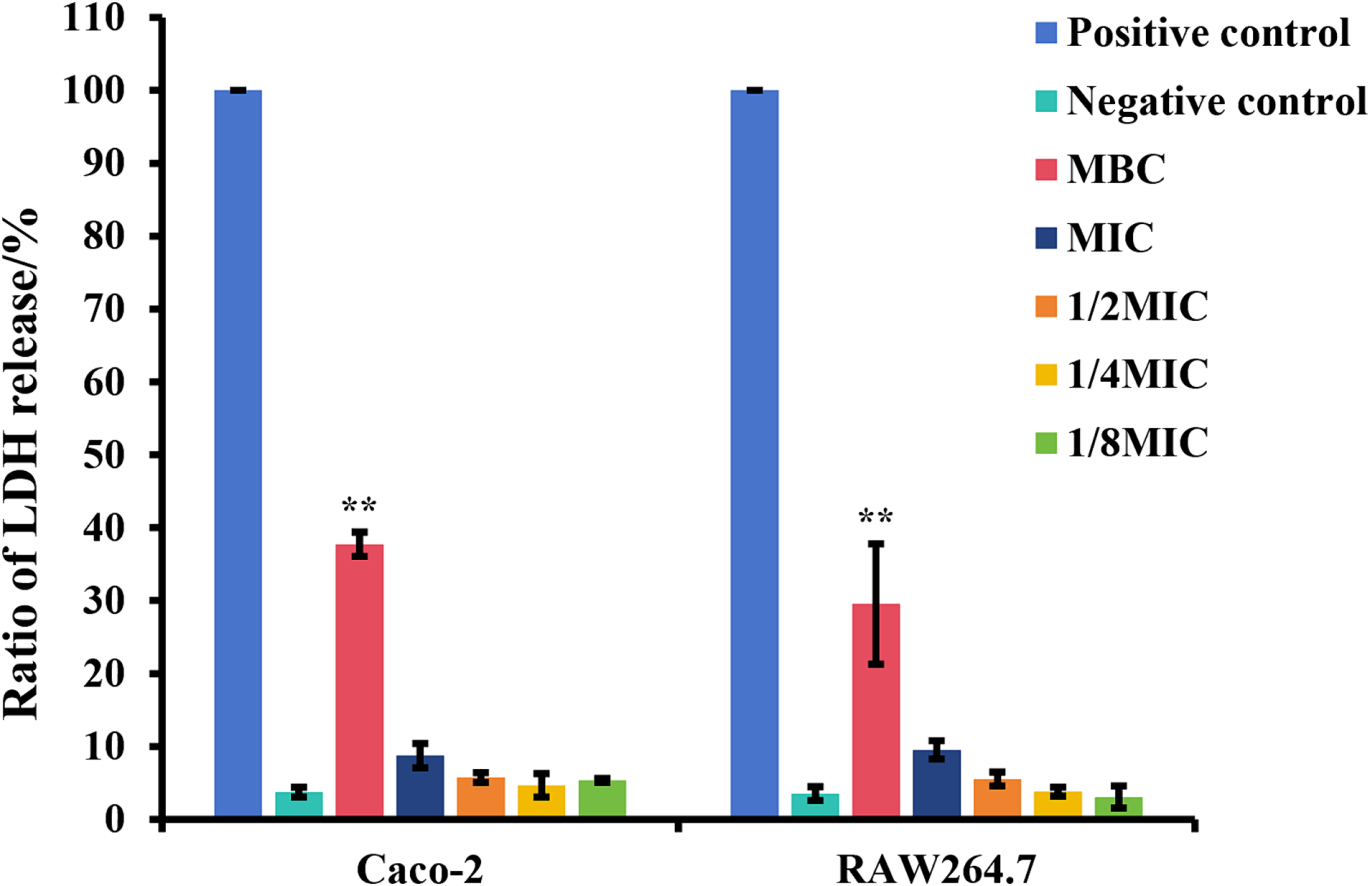
The ratio of lactate dehydrogenase (LDH) release of Caco-2 and RAW264.7 cells in the presence of glabridin at subinhibitory concentrations. Cytotoxicity was determined by the LDH method following a 6 h incubation with subinhibitory concentrations (1/8, 1/4, or 1/2 of the MIC), the MIC or MBC of glabridin. 0.2% Triton X-100 or DMEM only was used as the positive control and negative control, respectively. The experiments were repeated three times.

The cytotoxic effects of subinhibitory concentrations of glabridin on epithelial cells and macrophages were investigated via the LDH method following 6 h of treatment. As shown in Figure 6, different concentrations of glabridin (1/8, 1/4, or 1/2 of the MIC) had no toxic effect on Caco-2 and RAW264.7 cells (Figure 6). The results showed that subinhibitory concentrations of glabridin did not induce the release of LDH from Caco-2 and RAW264.7 cells. Concentrations of 1/8, 1/4, or 1/2 of the MIC were used for additional cell studies.

### 3.8 Subinhibitory concentrations of glabridin decrease the intracellular growth of *L. monocytogenes* in RAW264.7 cells

Gentamicin survival assays were performed to evaluate the influence of glabridin at subinhibitory concentrations on the intracellular growth of *L. monocytogenes* in RAW264.7 cells, as previously described. The results of the intracellular growth assay are illustrated in Supplementary Figure S3. After exposure to subinhibitory concentrations of glabridin at 1/4 or 1/2 of the MIC, the number of intracellular bacteria in RAW264.7 cells was much lower than that in the glabridin-untreated *L. monocytogenes* (Supplementary Figure S3). Glabridin inhibited the intracellular growth of *L. monocytogenes* in a concentration-dependent manner. However, treatment with glabridin at 1/8 of the MIC did not affect the intracellular growth of *L. monocytogenes* in RAW264.7 cells. This result suggested that subinhibitory concentrations of glabridin could improve *L. monocytogenes* clearance in RAW264.7 cells.

### 3.9 Effect of subinhibitory concentrations of glabridin on the ability of *L. monocytogenes* to trigger MET formation

**Figure 7:**
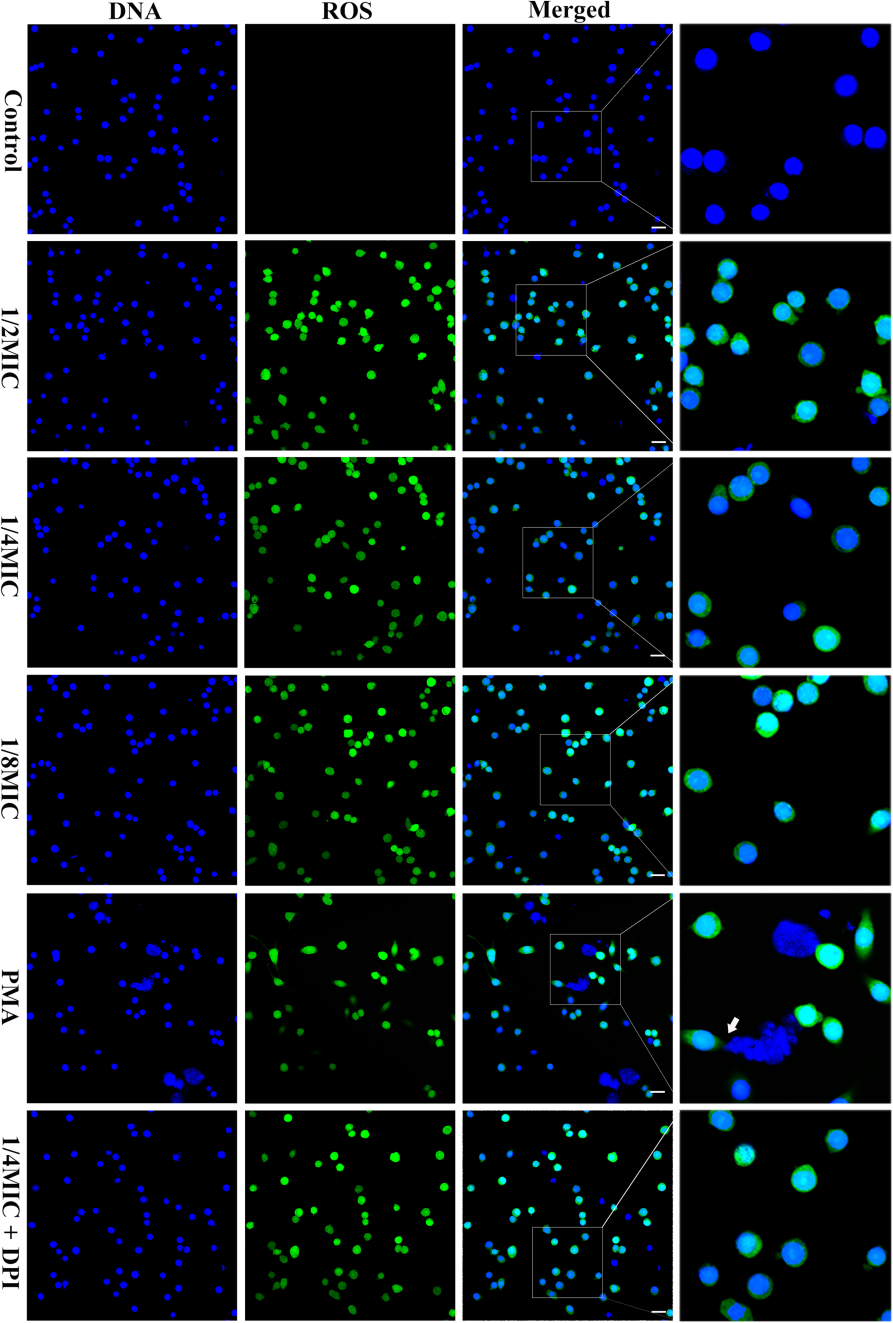
Glabridin and *L. monocytogenes* induced METs and intracellular ROS in RAW264.7 cells. RAW264.7 cells were infected with *L. monocytogenes* (MOI 10) and glabridin. After incubation for 2 h, Hoechst 33342 was added to stain the extracellular DNA. MET formation was observed using confocal laser scanning microscopy. PMA was used as the only positive control. Intracellular ROS levels in RAW264.7 cells were observed with DCFH-DA staining. The experiments were repeated three times. Scale bars: 20 μm. The white arrow represents METs.

We incubated RAW264.7 cells with glabridin (at 1/8, 1/4, or 1/2 of the MIC) for 2 h to examine the effect of glabridin on MET formation in macrophages. The fluorescence imaging analysis revealed that compared with the control treatment, treatment with PMA, an inducer of METs, could stimulate macrophages to release METs (Figure 7). The untreated macrophages did not release extracellular DNA. However, treatment with glabridin (1/8, 1/4, or 1/2 of the MIC) did not induce MET formation in macrophages (Figure 7). However, the presence of glabridin at the MBC significantly induced the release of extracellular DNA from macrophages (Supplementary Figure S4). In addition, we also observed intracellular ROS generation in macrophages exposure to glabridin (1/8, 1/4, or 1/2 of the MIC) (Figure 7). No significant difference in ROS production was detected among the glabridin treatments.

**Figure 8:**
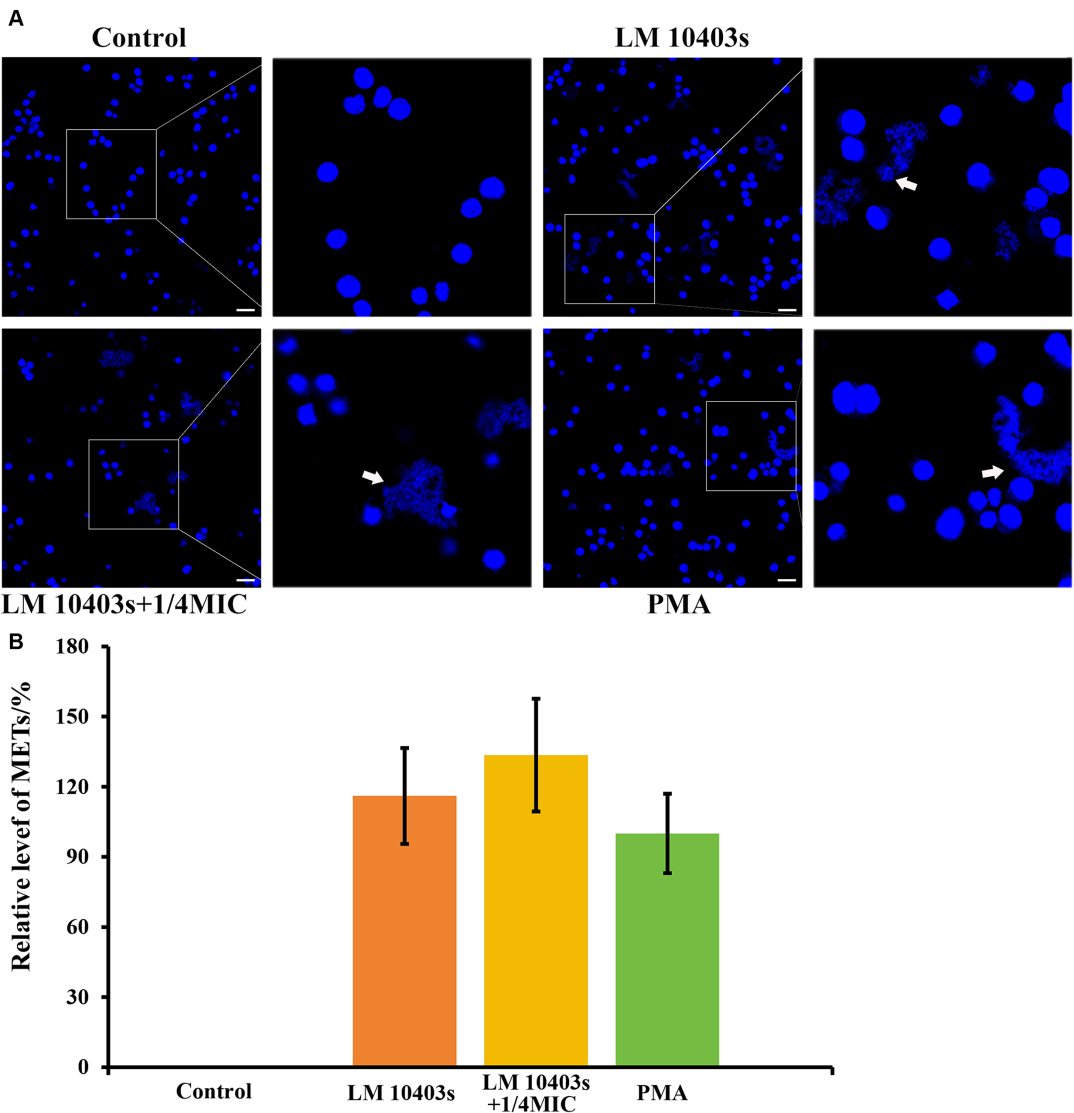
Effect of glabridin at subinhibitory concentrations on *L. monocytogenes* induced METs in RAW264.7 cells. RAW264.7 cells were infected with *L. monocytogenes* (MOI 10) and glabridin. After incubation for 2 h, Hoechst 33342 was added to stain the extracellular DNA. **(A)** MET formation was observed using confocal laser scanning microscopy. **(B)** Quantification of the MET formation by counting the percentage of RAW264.7 cells with METs. PMA was used as the only positive control. The MET formation in the PMA-treated macrophages was set as 100%. Data are represented as percentage of the PMA-treated group. The experiments were repeated three times. Scale bars: 20 μm. The white arrow represents METs.

Next, we asked ourselves whether subinhibitory concentrations of glabridin would have any effect on the ability of *L. monocytogenes*-stimulated macrophages to release METs. For this experiment, we incubated RAW264.7 cells with *L. monocytogenes* treated with glabridin for 2 h. Figure 8 shows that treatment with *L. monocytogenes* alone induced MET release from macrophages (Figure 8A). A similar phenomenon was also observed in which *L. monocytogenes* induced the release of extracellular reticulation structures after exposure to glabridin; however, the extent of MET formation was not significantly different from that of *L. monocytogenes* alone (Figure 8B). Based on these results, we showed that subinhibitory concentrations of glabridin did not affect the *L. monocytogenes*-induced MET release from macrophages.

### 3.10 Effect of subinhibitory concentrations of glabridin on the MET-mediated killing of extracellular bacteria by macrophages

**Figure 9:**
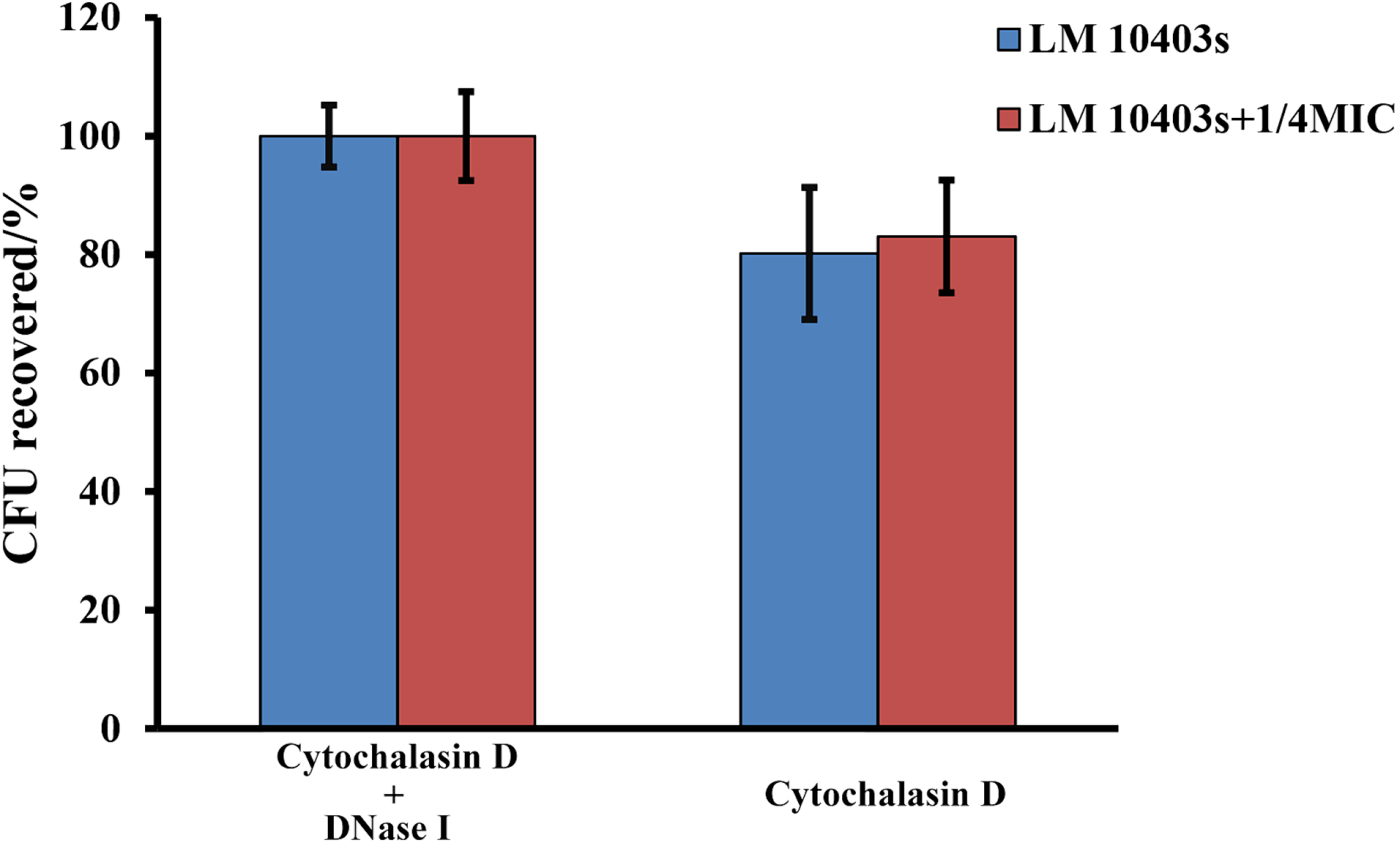
Effect of glabridin at subinhibitory concentrations on MET-mediated extracellular killing of macrophages against *L. monocytogenes*. RAW264.7 cells infected by *L. monocytogenes* were treated with subinhibitory concentrations of glabridin. Cytochalasin D and DNase I were added to inhibit phagocytosis and MET formation. The number of viable bacteria in the RAW264.7 cells was determined by CFU quantification. Pretreatment with cytochalasin D and DNase I was used as the 100% survival control group. The experiments were repeated three times.

The ET-mediated extracellular bactericidal effect is a novel defense mechanism of innate immune cells. *L. monocytogenes* was co-incubated with RAW264.7 cells in the presence of glabridin to determine whether subinhibitory concentrations of glabridin contribute to MET-mediated killing of extracellular bacteria by macrophages. We found that after a co-incubation with RAW264.7 cells and *L. monocytogenes* at 37°C for 2 h, *L. monocytogenes* was killed by macrophages compared with the control group (Figure 9). Efficient bacterial killing by macrophages was observed after the addition of glabridin. Cytochalasin D (10 μg/mL) was used to block phagocytosis to assess the effect of glabridin on extracellular *L. monocytogenes* clearance by METs. The results showed that the survival of *L. monocytogenes* treated with 1/4 of the MIC glabridin was similar to that of *L. monocytogenes* not treated with glabridin (Figure 9). This result showed that glabridin had no effect on the ability of macrophages to kill *L. monocytogenes* via METs.

## 4 Discussion

Research on the function of traditional Chinese medicine has become one of the hotspots in the research and development of antibacterial drugs. Licorice is a medicinal herb with a 2000-year history of application in traditional Chinese medicine. The most studied and documented benefits of glabridin from licorice are its anti-inflammatory properties, and its antibacterial activities have also been described, albeit less frequently, in the reported literature (Simmler et al., 2013; Ji et al., 2024). *L. monocytogenes* is a dangerous foodborne pathogen that poses a serious threat to public health and causes substantial economic losses for society. Subinhibitory concentrations of antibiotics are known to elicit multiple responses in bacteria. This study was performed to determine the effect of subinhibitory concentrations of glabridin on *L. monocytogenes*.

According to previous reports, the MICs of glabridin against pathogens vary from 1.562 to 31.3 μg/mL (Okada et al., 1989; Simmler et al., 2013; Zhang J. et al., 2023). The pathogens tested were *Enterococcus faecalis*, *Fusobacterium nucleatum*, *Bacillus subtilis*, *Staphylococcus aureus* (*S. aureus*), *Streptococcus mutans* (*S. mutans*), *Porphyromonas gingivalis* (*P. gingivalis*), *P. endodontalis*, *Prevotella intermedia*, *Mycobacterium smegmatis*, *Candida albicans* (*C. albicans*), *C. glabrata*, *C. utilis*, *Fusarium graminearum*, *Saccharomyces cerevisiae*, *Mucor pusillus*, *Aspergillus niger*, *Eimeria tenella*, and *Corynespora cassiicola* (Simmler et al., 2013; Zhang J. et al., 2023). Although the antimicrobial spectrum of glabridin *in vitro* has been relatively little studied, the active substances can promote non-specific immune function and enhance cellular immunity (Chirumbolo, 2016), which is then able to exert an anti-inflammatory effect on pathogen infection. The anti-inflammatory effects of glabridin have also been evaluated *in vitro* in microglia, promyelocytic cells, and dendritic cells, as well as *in vivo* in mice (Simmler et al., 2013). Glabridin ameliorates dextran sulfate sodium-induced colitis in mice by reducing colonic myeloperoxidase activity and the production of inflammatory mediators, such as nitric oxide, prostaglandin E2, and proinflammatory cytokines (Kwon et al., 2008).

In the present study, glabridin from Xinjiang licorice exhibited antimicrobial activity against *L. monocytogenes*, and the MIC and MBC were 31.25 μg/mL and 125 μg/mL, respectively. Okada et al. (1989) reported that glabridin had no antibacterial activity against the gram-negative bacteria, *E. coli* or *Pseudomonas aeruginosa*. However, treatment with glabridin at the MIC (12.5 μg/mL) exhibits antibacterial activity against *Helicobacter pylori* by inhibiting protein synthesis, DNA gyrase, and dihydrofolate reductase (Asha et al., 2013). Bombelli et al. (2023) reported that the MIC and MBC of glabridin against *L. monocytogenes* EGDe are 12.5 and 25 μg/mL, respectively. They also confirmed that temperature, pH, and oxygen were factors that influenced the antibacterial activity of glabridin against *L. monocytogenes*. Overall, glabridin exhibits only weak and non-specific antibiotic properties, and the antimicrobial properties of glabridin are mainly bacteriostatic. In addition to this particular activity, the antimicrobial mechanism of glabridin is still unclear. Compared with the control, treatment with glabridin at 1/8, 1/4, or 1/2 of the MIC did not limit the growth of *L. monocytogenes* in BHI at any time point during the incubation. In fact, glabridin at the MBC was sufficient to stimulate the disruption of the membrane integrity of *L. monocytogenes* (Bombelli et al., 2024). We then established subinhibitory concentrations of 1/8, 1/4, or 1/2 of the MIC of glabridin (i.e., 3.91, 7.81, and 15.63 μg/mL, respectively) that were used in subsequent experiments.

Previous studies have shown that swarming, swimming, twitching, gliding, and sliding are modes of bacterial movement (Kearns, 2010; Zegadlo et al., 2023). Swimming and swarming motility are two active forms of movement found in most bacteria, as observed in *L. monocytogenes* (Borges et al., 2012). In our study, we found a decrease in the swimming and swarming motility of *L. monocytogenes* in the presence of glabridin. Previous studies have shown that flagella are important drivers of bacterial motility. Interestingly, our study revealed that the number of flagella of *L. monocytogenes* did not change significantly in the presence of subinhibitory concentrations of glabridin. Therefore, from the TEM results, the reduced swimming and swarming motility were clearly not related to the flagellar production in *L. monocytogenes* cultured in the presence of glabridin. This result can be attributed to the loss of functionality of the flagellum (Inamuco et al., 2012). Swarming motility refers to multicellular surface movement in a fixed direction on a semisolid surface by the rotation of flagella. Swimming motility refers to individual movement in a fixed direction on a semisolid medium in aqueous environments by the rotation of flagella (Kearns, 2010; Wu et al., 2022). However, the mechanism by which glabridin affects swimming and swarming motility but not flagellar production is unclear. As shown in Figure 2, the effect of glabridin on the swarming motility of *L. monocytogenes* was greater than that on the swimming motility. This result may be related to the fact that bacterial swarming motility consumes more energy for flagellar activity than swimming motility (Poudel et al., 2019). However, extracellular ATP levels in *L. monocytogenes* were slightly decreased in the presence of glabridin, but the difference was not statistically significant. Furthermore, subinhibitory concentrations of antimicrobial agents reduce microbial pathogenicity by inhibiting the production of virulence factors because toxin secretion is usually co-regulated by swarming motility (Kearns, 2010). However, more experimental work is needed in order to support such claims.

Biofilms are complex surface-attached communities composed of extracellular polymeric substances (Muhammad et al., 2020). Most bacteria naturally grow in biofilms, which render the infection difficult to treat due to their high resistance to antibiotics and host immunity. Compared with their planktonic counterparts, bacteria in biofilms present up to a 1,000-fold increase in resistance to antimicrobial agents (Summer et al., 2022). Approximately 60% of foodborne illness outbreaks and 80% of human infections are likely related to microbial biofilms (Zafer et al., 2024). Biofilms could be promising therapeutic targets for the treatment of pathogenic bacterial infections. *L. monocytogenes* has a high capacity for biofilm formation on food and food-processing surfaces in food-processing environments. Plant-derived antimicrobial substances are generally considered safe, effective, and environmentally friendly for the prevention of foodborne diseases. These compounds have a negative effect on the biofilm formation of *L. monocytogenes*. Gallic acid treatment (at 5.8 mM) and resveratrol treatment (0.2–0.8 mM) reduced the overall biomass of *L. monocytogenes* biofilms (Ferreira and Domingues, 2016). Previous studies have shown that the minimum biofilm eradication concentrations of glabridin were 25, 50, and 25 μg/mL for *S. mutans*, *S. aureus*, and *P. gingivalis* biofilms, respectively (Tsukatani et al., 2022), but this effect has not been reported for *L. monocytogenes*. In our study, we found that 125 μg/mL glabridin exhibited a poor ability to eradicate *L. monocytogenes* biofilms. Furthermore, the spectrum of antibiofilm activities of glabridin has been described in relatively few articles. The formation of MRSA 4423 biofilms was completely reduced in the presence of glabridin at 6.25 μg/mL (1/2 of the MIC) (Gangwar et al., 2020). Moreover, 12 μg/mL (MIC) significantly inhibited the formation of *Aspergillus fumigatus* biofilms (Gao H. et al., 2022), and 12.5 μg/mL (2× MIC) significantly reduced the viability of *S. mutans* biofilms following a 2 h treatment (Vaillancourt et al., 2021). At 25 μg/mL, 11.2% of *E. faecalis* embedded in a biofilm was killed following 15 min of contact (Marcoux et al., 2020), whereas at 1/4 of the MIC (3.125 μg/mL), biofilm formation was not attenuated after 30 min of contact (Grenier et al., 2020). Moreover, 1/4 of the MIC (128 μg/mL) and 1/2 of the MIC significantly decreased biofilm formation by multidrug-resistant *Acinetobacter baumannii* by 19.98 and 27.43%, respectively (Lin et al., 2023).

Antibiotics have been shown to decrease the abundance of microorganisms in biofilms. Subinhibitory concentrations of allicin have been shown to inhibit the formation of *C. albicans*, *S. epidermidis*, and uropathogenic *E. coli* biofilms (Yang et al., 2016). However, exposure to subinhibitory concentrations of antibiotics has been found to induce biofilm formation in several pathogenic species (Jolivet-Gougeon and Bonnaure-Mallet, 2014). The MIC for *Clostridioides difficile* (*C. difficile*) increased following exposure to subinhibitory concentrations of metronidazole. Doan et al. (2022) also reported that the production of *C. difficile* biofilms was induced by exposure to subinhibitory concentrations of metronidazole. He et al. (2022) reported that subinhibitory concentrations of ethanol (1/4 of the MIC, 2.5%; 1/2 of the MIC, 5.0%) were able to stimulate biofilm formation in *Salmonella enteritidis*. Park et al. (2020) documented that subinhibitory concentrations of chlorhexidine and mupirocin promoted biofilm formation in the clinical methicillin-resistant *S. aureus* isolates. Accordingly, this study evaluated the effect of subinhibitory concentrations of glabridin on *L. monocytogenes*. At 1/8, 1/4, or 1/2 of the MIC, glabridin had no effect on biofilm formation by *L. monocytogenes*. Flagella are very important for biofilm formation in several bacterial species. As an adhesive structure, flagella participate in the initial phase of *L. monocytogenes* biofilm formation (Vatanyoopaisarn et al., 2000). In this study, the flagella of *L. monocytogenes* did not change upon exposure to subinhibitory concentrations of glabridin. In addition, exposure to subinhibitory concentrations of antibiotics can stimulate the formation of factors such as toxins and adhesins (Gao P. et al., 2022).

Listeriolysin O (LLO), encoded by the *hly* gene, is recognized as a *Listeria*-specific hemolysin. Screening natural compounds as inhibitors of LLO is a hot topic in the research of antimicrobial agents against *L. monocytogenes* infection. Fisetin, a natural flavonoid from vegetables and fruits, significantly decreases the hemolytic activity of an *L. monocytogenes* culture supernatant (Wang et al., 2015). Glabridin is an isoflavan isolated from licorice root extract. Consistent with previous studies, our results indicate that glabridin was more effective at inhibiting the hemolytic activity of *L. monocytogenes*. Jiang et al. (2022) reported that the hemolytic activity of *L. monocytogenes* 10403S was almost completely inhibited upon exposure to 1/4 of the MIC of phenyllactic acid. Previous studies have shown that LLO is an essential virulence determinant for *L. monocytogenes* to escape from phagosomes (Nguyen et al., 2019). In our study, we analyzed the expression of the *hly* gene in *L. monocytogenes* exposed to glabridin using qRT-PCR. The qRT-PCR results revealed that the expression of the *hly* gene in *L. monocytogenes* treated with glabridin at 1/2 and 1/of the MIC was reduced significantly by 2.9- and 2.4-fold, respectively, compared to that in the untreated *L. monocytogenes*. Similarly, LLO secretion by *L. monocytogenes* 10403S was reduced by subinhibitory concentrations of phenyllactic acid (Jiang et al., 2022). In addition, Hou et al. (2023) reported that a cinnamon twig extract can effectively inhibit the hemolysis of LLO without inhibiting the growth of *L. monocytogenes*.

Pathogenic bacterial adhesion to host cell surfaces is the first and crucial step for the successful establishment of survival and replication during infection (Birhanu et al., 2018). However, the presence of glabridin at subinhibitory concentrations did not alter the adherence of *L. monocytogenes* to epithelial cells in this study (Supplementary Figure S5). Although the primary function of flagella is not to mediate adhesion, an impaired motility function conferred by flagella results in the reduced adhesion of pathogens (Zhai et al., 2024). One possibility is that the subinhibitory concentration of glabridin did not damage the flagella production in *L. monocytogenes*. The survival and replication of cells are well established as important virulence factors for pathogens. Macrophages play an important role in anti-inflammatory processes and pathogen clearance and serve as a crucial role in shaping both innate and adaptive immune responses. Rosenblat et al. (1999) confirmed that an incubation of macrophages (J774.1A) with glabridin (20 μM) for 20 h at 37°C was not cytotoxic to the cells. Consistent with this finding, we showed that subinhibitory concentrations (1/8, 1/4, or 1/2 of the MIC) of glabridin had no toxic effect on RAW264.7 cells in the present study. Notably, we observed that glabridin inhibited the intracellular growth of *L. monocytogenes* in a concentration-dependent manner. A previous study by Rosenblat’s laboratory showed that 60 and 40% of the glabridin was localized in the macrophage membrane and the cytosol, respectively (Rosenblat et al., 1999). Although subinhibitory concentrations of glabridin did not directly affect the growth rate of *L. monocytogenes*, these concentrations were more effective at inhibiting the hemolytic activity of *L. monocytogenes*. Hemolysin is responsible for the lysis of macrophage lysosomes (Okunnu and Berg, 2019). LLO produced by *L. monocytogenes* leads to lysis of the phagosomal membrane in macrophages, subsequently allowing *L. monocytogenes* to escape into the cytoplasm, where it is able to replicate and participate in cell–cell spread (Portnoy et al., 1988; Gedde et al., 2000; Nguyen et al., 2019). Moreover, glabridin has a notably distinctive mechanism of inhibiting the inflammatory response (Shin et al., 2023). Shin et al. (2023) reported that synthetic glabridin derivatives exert strong anti-inflammatory effects on LPS-induced RAW264.7 cells by inhibiting the MAPK and NF-κB pathways. Kang et al. (2005) documented that glabridin inhibits NO production and iNOS gene expression by blocking NF-κB/Rel activation in RAW264.7 cells. Glabridin reverses changes in amino acid, energy, and lipid metabolism during the LPS-induced inflammatory process in RAW264.7 cells (Liu et al., 2017). In addition, tiliroside, a flavonoid present in edible plants, may inhibit a major signaling pathway upstream of glycolytic enzymes (Zhuang et al., 2021). These effects may be beneficial for reducing macrophage damage and subsequent bacterial dissemination.

ETs are composed of extracellular DNA supplemented with various protein substances and have been shown to be a unique defense strategy for the immune system (Liao et al., 2022). Vertebrate innate immune cells, including neutrophils, mast cells, eosinophils, and macrophages, are capable of forming ETs, as are invertebrate and plant cells. Previous studies have shown that various pathogens and their products and chemical reagents can stimulate immune cells to release METs (Cubillo-Martinez et al., 2022). In our present study, we observed that subinhibitory concentrations of glabridin did not trigger extracellular DNA release from RAW264.7 cells. In fact, glabridin at the MBC was sufficient to stimulate macrophages to release METs. NADPH oxidase-dependent ROS production is thought to be important for ET formation (Azzouz and Palaniyar, 2022). Several studies have shown that the NADPH oxidase-independent pathway is another key mechanism of NET formation based on the death of neutrophils (Khan and Palaniyar, 2017). Here, we found that glabridin-induced ROS production was similar to that induced by PMA, and ROS production was detected in the presence or absence of DPI (an NADPH oxidase inhibitor).

*L. monocytogenes* has been shown to trigger the formation of METs in macrophages (Cheng et al., 2015). Next, we examined the effect of glabridin on MET formation induced by *L. monocytogenes*. The amount of METs formed by macrophages infected with *L. monocytogenes* was not altered by the addition of glabridin. No obvious synergistic effects of glabridin and *L. monocytogenes* on enhancing MET formation were detected. The mechanisms by which glabridin works positively or negatively on NADPH oxidase and ROS have not yet been clearly described. Rosenblat et al. (1999) reported that glabridin has no direct inhibitory effect on the active NADPH oxidase; however, it can inhibit the cellular processes that lead to NADPH oxidase activation. Glabridin attenuates ROS production induced by 2-deoxy-D-ribose in the mouse osteoblastic cell line MC3T3-E1 (Kim et al., 2013), UVB-irradiation of human keratinocytes, and NaIO_3_ in retinal pigment epithelial ARPE-19 cells (Veratti et al., 2011). Thus, glabridin prevents direct and indirect DNA damage to avoid activating cell death pathways (Aung et al., 2020). ETosis is a form of inflammatory cell death that occurs following ET formation in immune cells. However, Zhang et al. (2022) reported that intracellular ROS levels were dose-dependently increased in the prostate cancer cell lines DU-145 and LNCaP after glabridin treatment. ETs are considered a novel bactericidal mechanism that differ from phagocytosis (Arabi et al., 2023). However, glabridin did not affect the killing of extracellular bacteria by macrophages via METs, as no obvious synergistic effect on MET formation induced by *L. monocytogenes* was detected.

## 5 Conclusion

In summary, we found that subinhibitory concentrations of glabridin did not affect the growth, morphology, flagellar production, or biofilm formation of *L. monocytogenes* but inhibited motility and decreased hemolytic activity. At subinhibitory concentrations, glabridin did not affect the ability of *L. monocytogenes* adhere to Caco-2 cells but decreased its intracellular growth in RAW264.7 cells. Glabridin induced ROS production in macrophages but did not affect MET formation, the capacity of *L. monocytogenes* to trigger METs, or extracellular killing by METs. These findings provide valuable insights into the further application of glabridin as an alternative antimicrobial agent to inhibit *L. monocytogenes*.

## Data availability statement

The raw data supporting the conclusions of this article will be made available by the authors, without undue reservation.

## Ethics statement

Ethical approval was not required for the studies on humans in accordance with the local legislation and institutional requirements because only commercially available established cell lines were used. Ethical approval was not required for the studies on animals in accordance with the local legislation and institutional requirements because only commercially available established cell lines were used.

## Author contributions

CL: Conceptualization, Methodology, Investigation, Writing – original draft, Writing – review & editing, Project administration, Funding acquisition. CY: Data curation, Methodology, Writing – review & editing. JG: Methodology, Writing – review & editing. MG: Methodology, Writing – review & editing.
