# Supplementary figures and images for: Subinhibitory concentrations of glabridin from Glycyrrhiza glabra L. reduce Listeria monocytogenes motility and hemolytic activity but do not exhibit antimicrobial activity
Source: Front Microbiol. 2024 Jul 17;15:1388388. doi: 10.3389/fmicb.2024.1388388 (PMC11288822; doi:10.3389/fmicb.2024.1388388)

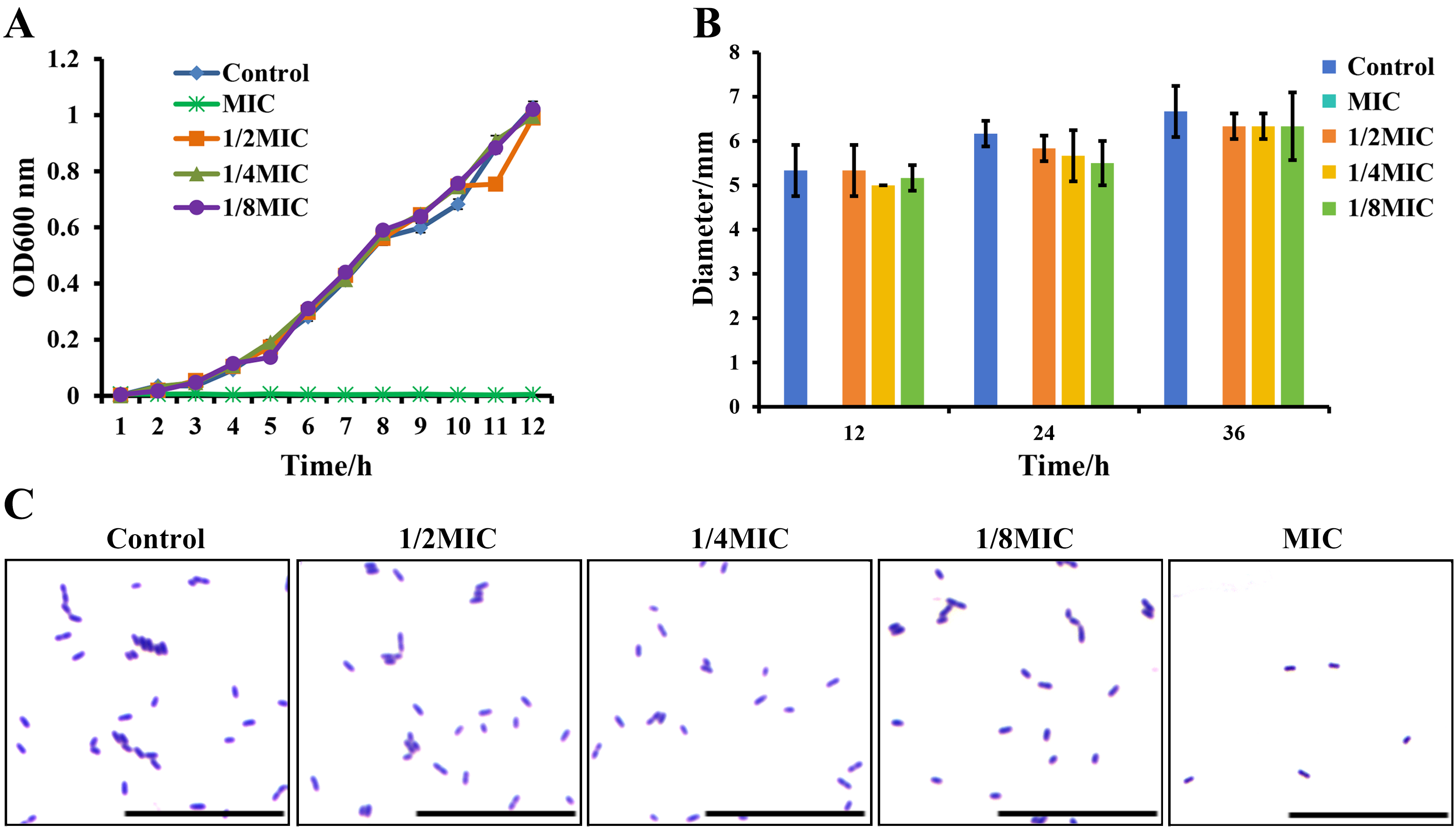

Supplement: Supplementary file 1 [file Image_1.TIF]

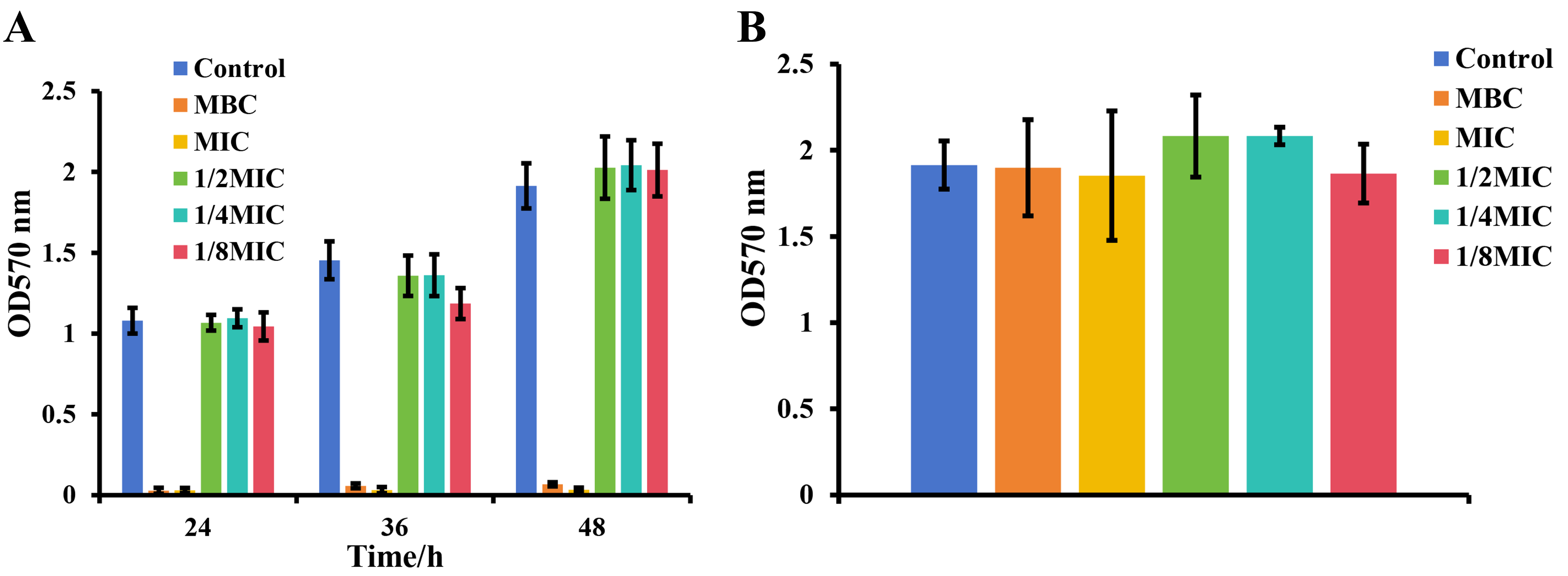

Supplement: Supplementary file 2 [file Image_2.TIF]

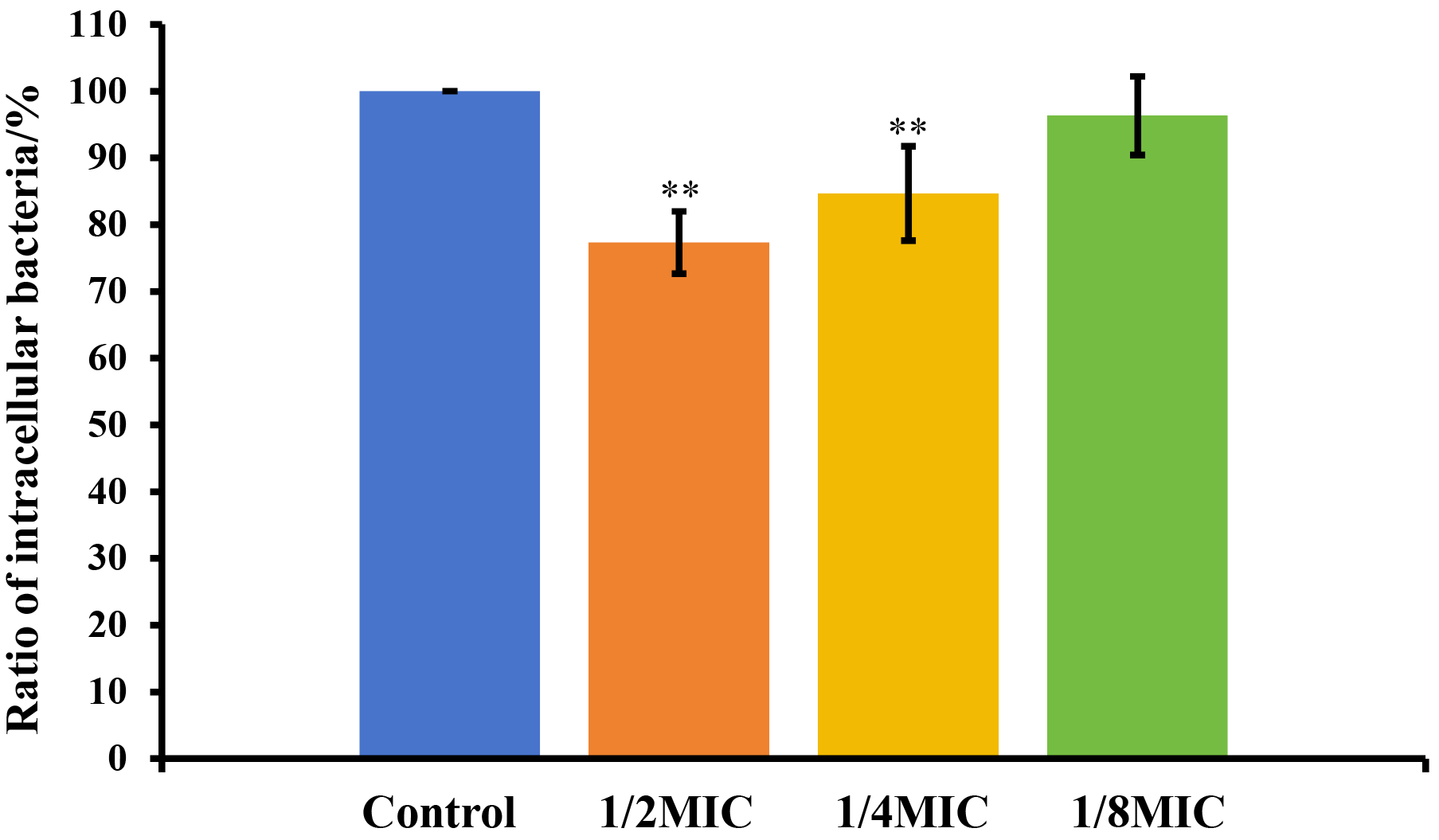

Supplement: Supplementary file 3 [file Image_3.TIF]

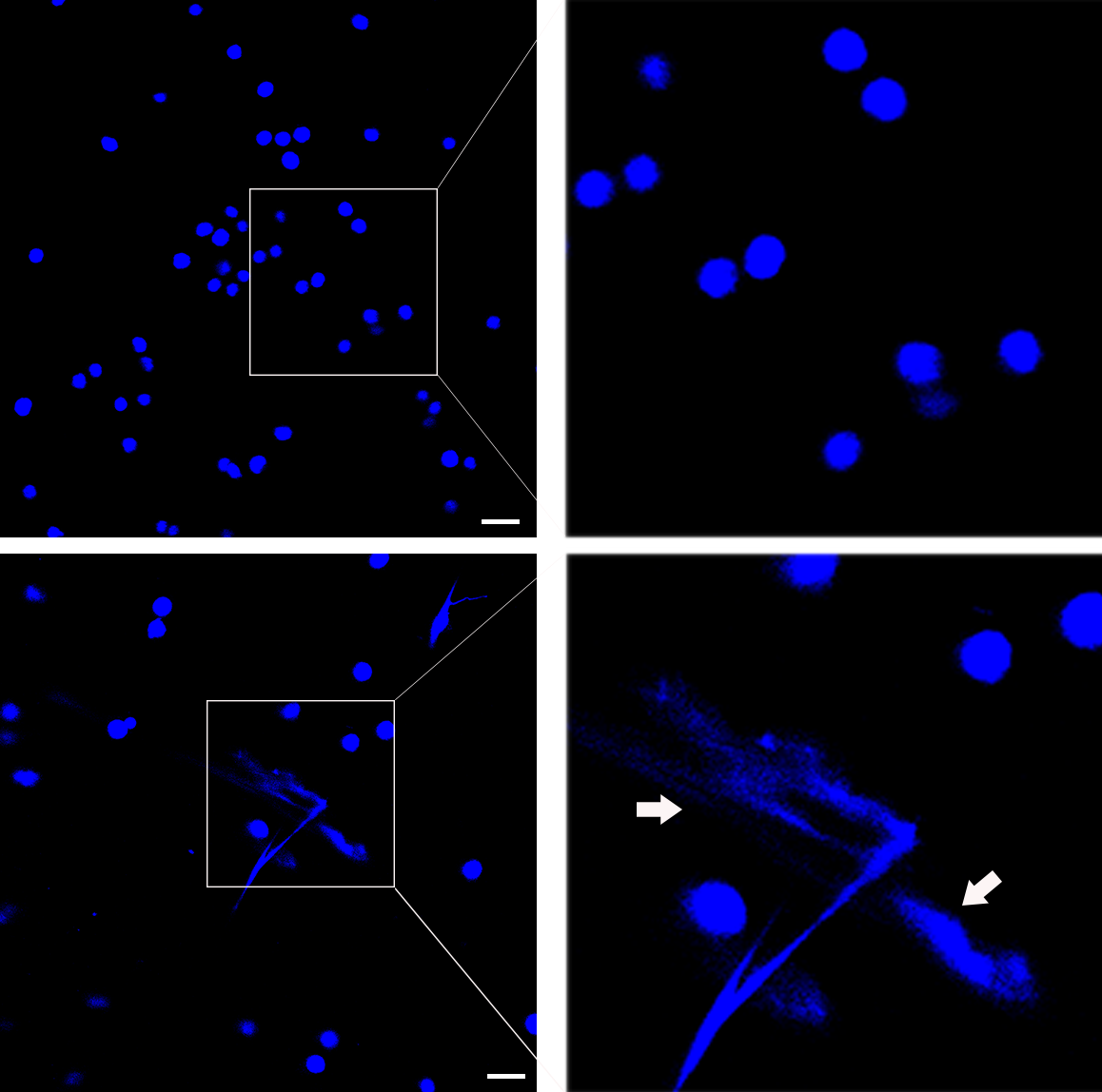

Supplement: Supplementary file 4 [file Image_4.TIF]

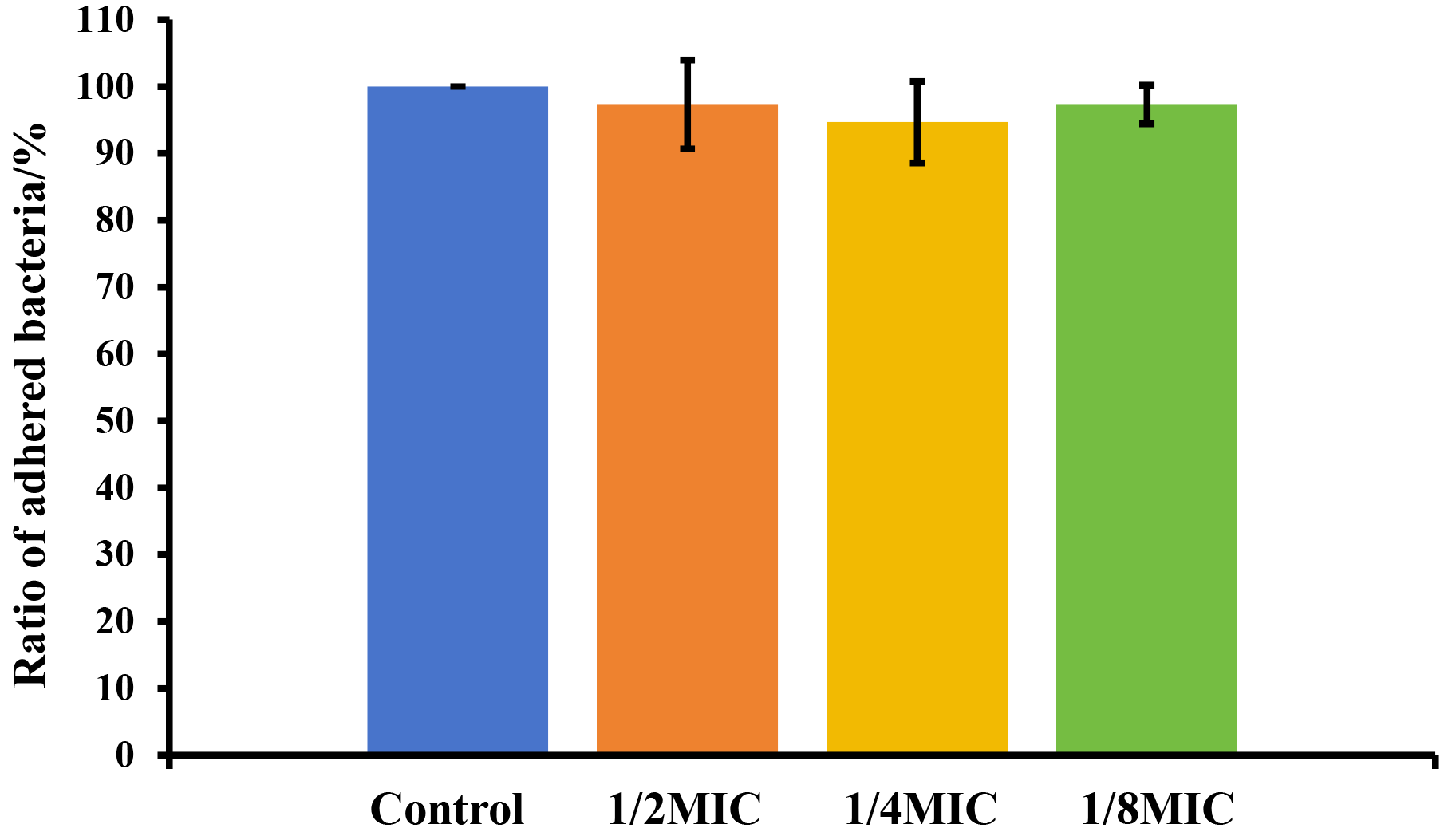

Supplement: Supplementary file 5 [file Image_5.TIF]
